# Real-world safety of ixekizumab: a disproportionality analysis using the FDA adverse event reporting system and the VigiAccess databases

**DOI:** 10.3389/fmed.2025.1652401

**Published:** 2025-10-13

**Authors:** Kaidi Zhao, Xuanfeng Tan, Miao Qin, Qinxiao Li, Qinqin Meng, Ying Liu, Jing Li, Jiashu Liu

**Affiliations:** 1 Department of Dermatology, The Second Affiliated Hospital of Xi'an Jiaotong University, Xi'an, China; 2 Department of Dermatology, Shanghai Sixth People's Hospital Affiliated to Shanghai Jiao Tong University School of Medicine, Shanghai, China; 3 Department of Dermatology and Aesthetic Medicine, Honghui Hospital, Xi’an Jiaotong University, Xi’an, China; 4 Department of Dermatology, Xi’an Children’s Hospital, National Regional Children’s Medical Center (Northwest), Xi’an, China

**Keywords:** ixekizumab, biologics, psoriasis, FAERS, disproportionality analysis, adverse events

## Abstract

**Introduction:**

Ixekizumab is a biologic agent primarily indicated for the treatment of moderate to-severe plaque psoriasis. This study aimed to evaluate the post-marketing safety profile of ixekizumab by analyzing adverse event (AE) reports retrieved from the Food and Drug Administration Adverse Event Reporting System (FAERS) database and VigiAccess databases.

**Methods:**

Four disproportionality analysis methods were employed in this study to detect positive signals associated with ixekizumab, including Reporting Odds Ratio (ROR), Proportional Reporting Ratio (PRR), Bayesian Confidence Propagation Neural Network (BCPNN), and Multi-item Gamma Poisson Shrinker (MGPS). Sensitivity analyses were conducted to ensure the robustness of the findings. Additionally, the time to onset of AEs was further analyzed.

**Results:**

In the FAERS databases and VigiAccess databases, 72,847 AE reports in total. Commonly reported AEs included injection site reactions, hypersensitivity reactions, fungal infections, upper respiratory tract infections, and inflammatory bowel disease. In addition, several unexpected AEs were identified, such as cellulitis, ear infection, bronchitis, herpes zoster, tooth infection, diverticulitis, kidney infection, and gastroenteritis viral. Sensitivity analysis further confirmed the robustness of these findings. Notably, 41.1% of the AEs occurred within the first month after treatment initiation.

**Discussion:**

This study confirmed several known AEs and identified some unexpected AEs, providing preliminary safety insights to guide clinicians in the safe use of ixekizumab in clinical practice. It is important to note that findings from spontaneous adverse event reporting systems are hypothesis-generating and may be limited by underreporting, variable reporting quality, and confounding factors.

## 1 Introduction

Psoriasis, a chronic inflammatory dermatological condition, impacts approximately 125 million people globally (1). It is characterized by extensive erythematous plaques and scales on the skin, frequently accompanied by other chronic disorders such as arthritis, hypertension, diabetes, and obesity (2). Psoriatic arthritis is a heterogeneous, chronic inflammatory musculoskeletal disorder affecting approximately 30% of individuals with psoriasis. Notably, more than half of psoriatic arthritis patients develop a progressive and erosive form of the disease, which ultimately leads to functional impairment (3, 4). Previous research has already linked psoriasis and psoriatic arthritis with significant psychological burdens, including depression, unhealthy risk behaviors, and negative body image (5). Research indicates that psoriasis and psoriatic arthritis share a common underlying pathophysiological mechanism involving the disorder of the immune system, particularly the impact of cytokines such as IL-17 (2, 3, 6). Psoriatic lesions are characterized by the infiltration of a substantial number of inflammatory cells, and the IL-17 signaling pathway is pivotal in the disease’s pathogenesis (6).

Ixekizumab (IXE) is a monoclonal antibody that selectively targets IL-17A, an inflammatory cytokine that plays a critical role in the pathogenesis of psoriasis. The drug received approval from the FDA for the treatment of psoriasis in 2016, followed by its approval for psoriatic arthritis in 2017 (7). Various clinical trials have confirmed the efficacy and safety of IXE for the treatment of psoriasis (8, 9). Data from multiple clinical trials indicate that IXE treatment demonstrates good long-term tolerability in adult patients with plaque psoriasis, psoriatic arthritis, and axial spondyloarthritis. Numerous randomized clinical trials and real-world studies have consistently demonstrated that ixekizumab (IXE) is both effective and safe for the treatment of psoriasis, with the majority of associated adverse events being mild to moderate in severity. Additionally, the incidences of serious AE and discontinuations due to AEs were low, with these rates remaining consistently low throughout the five-year period (9–11). However, as the use of IXE increases across diverse patient populations, with greater variability in dosing frequency and duration, and considering that psoriasis typically requires long-term treatment, identifying unexpected rare or serious AEs, late complications, and previously unassessed occurrences is crucial.

FAERS is a powerful database that collects and monitors AE reports related to drugs and biologics. FAERS provides a valuable resource for assessing the safety of medications in a broader patient population, especially those who were not included in clinical trials. Due to its extensive and accessible real-world data, an increasing number of researchers have used the FAERS database to assess the safety of drugs (12, 13). VigiAccess, a global pharmacovigilance database managed by the World Health Organization, compiles AE reports from a wide range of countries and regions, offering a comprehensive, worldwide view of drug safety data (14). This study aimed to evaluate the real-world safety profile of IXE by mining drug AEs reported in the VigiAccess and FAERS databases, with a focus on identifying unexpected safety signals and providing preliminary safety data for the long-term use of this drug.

## 2 Methods

### 2.1 Data source and study design

The data for this study were sourced from the VigiAccess database, encompassing its entire history up until 8 September 2025, as well as the FAERS database, covering the period from the first quarter of 2016 to the third quarter of 2024.

FAERS database aggregates spontaneous AE reports from healthcare providers, patients, and manufacturers. The specific information categories are classified as follows: demographic and administrative information (DEMO), drug information (DRUG), adverse drug reaction information (REAC), patient outcome information (OUTC), reporting source information (RPSR), date of treatment initiation and end date of reported medication (THER), and medication administration indications (INDI). The seven datasets were merged using the primary parameter to facilitate further analysis.

**Figure 1:**
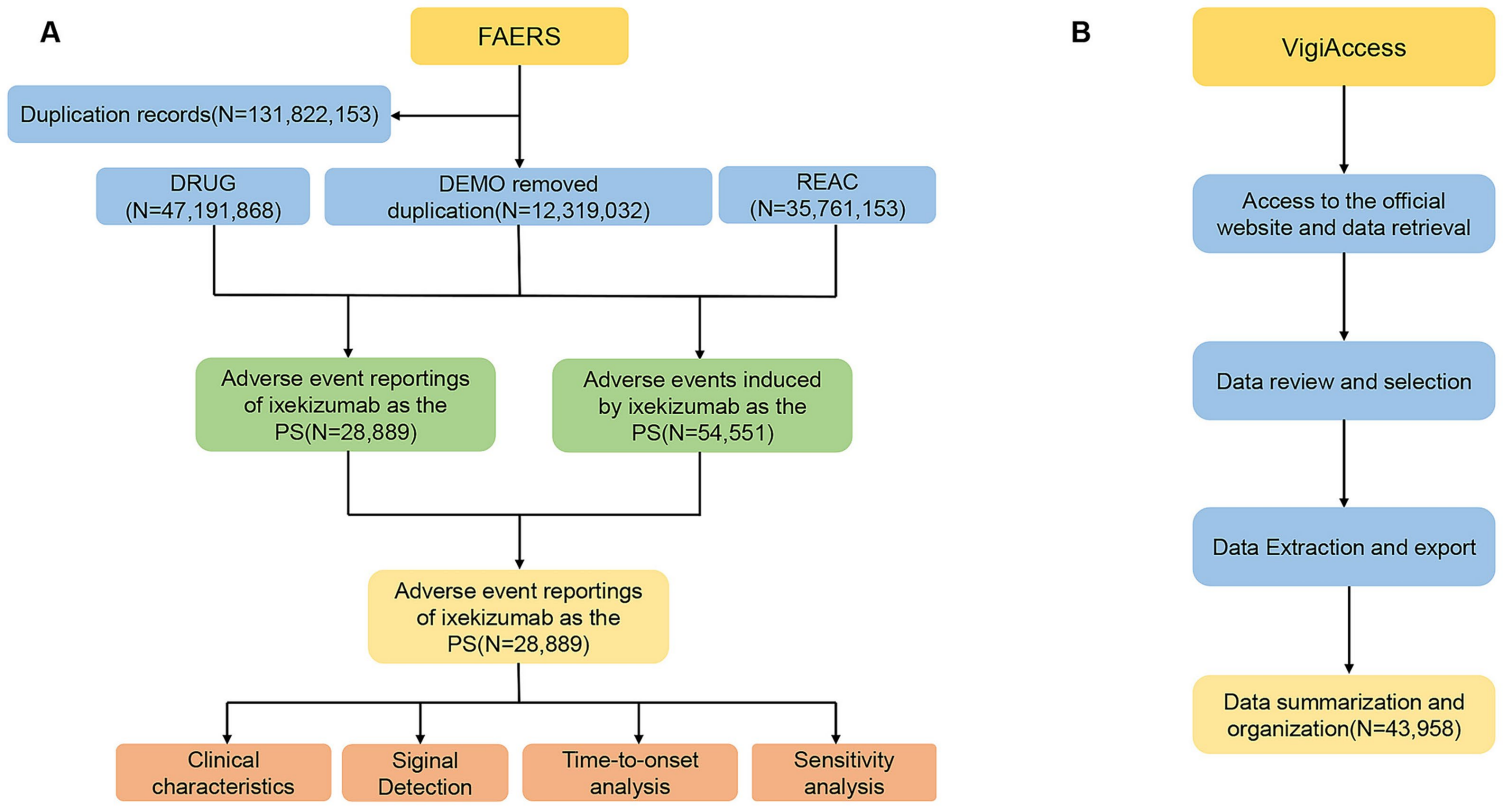
Flow diagram of the selection process for ixekizumab-related adverse events from FAERS and VigiAccess. **(A)** Data identification, extraction, and processing in FAERS. **(B)** Data identification, extraction, and processing in VigiAccess.

We removed duplicate reports from the data using Case Identifiers (CASEIDs), FDA Receipt Date (FDA_DT), and Primary Identifiers (PRIMARYID). In accordance with FDA recommended practices, when two reports share the same CASEID, the report with the later FDA_DT is retained; when both CASEID and FDA_DT match, the report with the highest PRIMARYID is retained. Deduplication of AE reports prior to disproportionality analysis is crucial for ensuring the accuracy of the analysis. Additionally, we excluded irrelevant AEs, such as those unrelated to the drug (e.g., drug ineffectiveness and treatment discontinuation) or events inherently associated with the disease itself (e.g., psoriasis, psoriatic arthritis, erythema). The details of data identification, extraction, and processing are depicted in Figure 1.

Furthermore, VigiAccess data were collected from https://www.vigiaccess.org, encompassing various age groups, genders, reporting years, and geographic regions. Searches were conducted using generic names, and AEs were described according to the System Organ Class (SOC) and Preferred Term (PT) of the Medical Dictionary for Regulatory Activities (MedDRA, version 27.1). This approach aims to enhance the credibility of the association between IXE and AEs.

### 2.2 Statistical analysis

We employed disproportionality analysis methods to quantify unexpected signals of IXE-associated AEs. The specific algorithms included ROR, PRR, BCPNN, and MGPS (15–17). We define a positive signal as meeting the criteria for positive according to all four algorithms. Supplementary Tables 1, 2 provide the criteria for positive signals and equations used for the aforementioned algorithms. We also conducted a sensitivity analysis by excluding the drugs most commonly co-administered with IXE. Additionally, we used the Weibull distribution to analyze changes in AE incidence over time and evaluated the time-to-event onset comprehensively using median, quartiles, and Weibull testing. The Weibull distribution include shape parameter (*β*) and scale parameter (*α*), which can further explain the trend of AEs and describe the characteristic time of occurrence. All analyses were performed using R software version 4.2.2.

## 3 Results

### 3.1 Baseline characteristics

In this study, the FAERS and VigiAccess databases reported 28,889 and 43,958 AE reports, respectively, associated with IXE, for which IXE was considered the primary suspected drug responsible for the adverse reactions. The demographic characteristics are shown in Table 1. Among the AE reports, females accounted for a higher proportion than males in both databases. With respect to age distribution, the 18–65 years age group represented the largest demographic, comprising 41.6% of reported cases in the FAERS database and 48.7% in the VigiAccess database. In both databases, the majority of reports originated from the Americas.

**Table 1:** Clinical characteristics of IXE adverse event reports from the FAERS and VigiAccess database.

Characteristics	FAERS	VigiAccess
Number of event reports	28,889	43,958
	n (%)	
Gender		
Male	10,400(36%)	16,527(37.6%)
Female	16,050(55.6%)	24,279(55.2%)
Miss	2,439(8.4%)	3,152(7.2%)
Age(years)		
Median (IQR)	53(42,62)	
<18	255(0.9%)	364(0.8%)
18–64	12,004(41.6%)	21,416(48.7%)
≥65	2,522(8.7%)	4,338(9.7%)
Miss	14,108(48.8%)	17,920(40.8%)
Reporting year		
2016	360(1.2%)	19(0.04%)
2017	2020(7.0%)	1,601(3.64%)
2018	3,583(12.4%)	3,678(8.37)
2019	4,487(15.5%)	7,373(16.77%)
2020	3,138(10.9%)	4,149(9.44%)
2021	3,598(12.5%)	5,156(11.73%)
2022	4,529(15.7%)	6,713(15.27%)
2023	4,036(14%)	6,322(14.38%)
2024	3,138(10.9%)	6,082(13.84%)
2025		2,865(6.52%)
Geographical distribution		
Americas	27,081(93.7%)	28,652(65.2%)
Europe	439(1.5%)	12,375(28.1%)
Asia	181(0.6%)	2,509(5.7%)
Africa		255(0.6%)
Oceania		167(0.4%)
Miss	1,188(4.2%)	
Reporter		
Consumer	15,673(54.3)	
Healthcare professional	3,434(11.9%)	
Other health-professional	3,112(10.8%)	
Physician	1822(6.3%)	
Pharmacist	1,559(5.4%)	
Lawyer	1	
Missing	3,288(11.4%)	

IQR, interquartile range.

### 3.2 Distribution of AEs at the system organ class level

**Figure 2:**
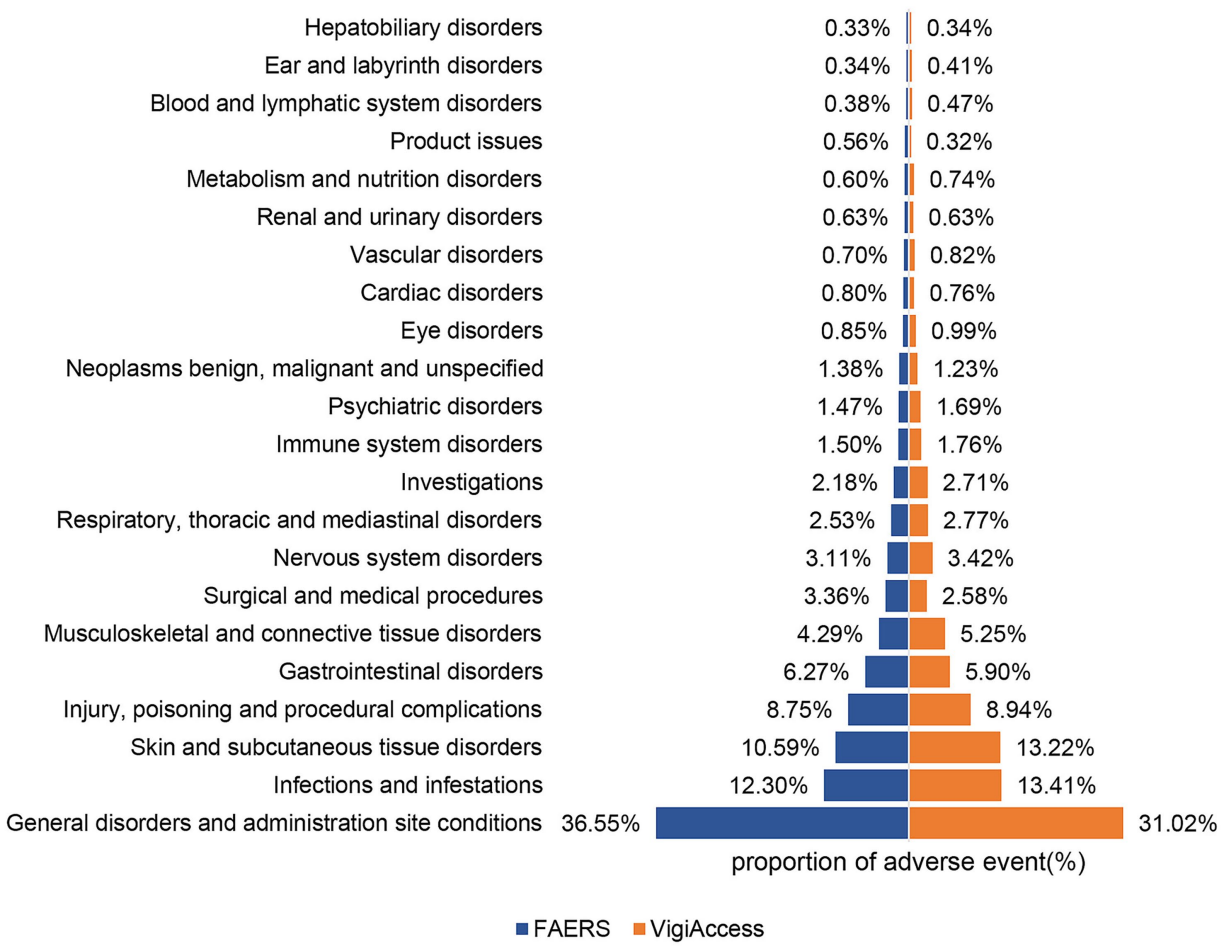
Proportions of AEs by system organ class for ixekizumab based on data from FAERS and VigiAccess.

Table 2 shows the distribution and signal values of IXE-related AEs at the SOC level. Among the IXE-induced AEs, “infections and infestations,” “general disorders and administration site conditions,” and “surgical and medical procedures” met the criteria of all four algorithms. “Skin and subcutaneous tissue disorders” and “immune system disorders” met the criteria of the ROR and BCPNN algorithms. The distribution of AEs at the SOC level is shown in Figure 2. In the VigiAccess database, the top 5 SOCs for IXE are General disorders and administration site conditions (31.0%), Infections and infestations (13.4%), Skin and subcutaneous tissue disorders (13.2%), Injury, poisoning and procedural complications (8.9%), Gastrointestinal disorder (5.9%), with detailed results presented in Supplementary Table 3. In summary, when AE signals for IXE are categorized by SOC, General disorders and administration site conditions, Infections and infestations, and Skin and subcutaneous tissue disorders consistently emerge as the top three SOC categories for the drug in both the FAERS and VigiAccess databases.

**Table 2:** Signal strength of ixekizumab AEs across system organ classes (SOC) in the FAERS database.

SOC	Case numbers	ROR(95%CI)	PRR(χ^2^)	EBGM(EBGM05)	IC(IC025)
Infections and infestations*	6,712	2.42 (2.36–2.48)	2.24 (4882.58)	2.24 (2.19)	1.16 (1.13)
General disorders and administration site conditions*	19,940	2.66 (2.61–2.7)	2.05 (13022.97)	2.05 (2.02)	1.03 (1.01)
Investigations	1,188	0.36 (0.34–0.38)	0.38 (1307.97)	0.38 (0.36)	−1.41 (−1.5)
Respiratory, thoracic and mediastinal disorders	1,379	0.53 (0.51–0.56)	0.55 (543.34)	0.55 (0.52)	−0.87 (−0.95)
Skin and subcutaneous tissue disorders*	5,777	1.95 (1.89–2)	1.85 (2367.2)	1.84 (1.8)	0.88 (0.84)
Gastrointestinal disorders	3,419	0.74 (0.72–0.77)	0.76 (288.82)	0.76 (0.74)	−0.4 (−0.45)
Immune system disorders*	818	1.23 (1.15–1.32)	1.23 (34.21)	1.22 (1.16)	0.29 (0.19)
Blood and lymphatic system disorders	206	0.22 (0.19–0.26)	0.23 (554.33)	0.23 (0.2)	−2.14 (−2.34)
Nervous system disorders	1,696	0.38 (0.36–0.4)	0.4 (1673.1)	0.4 (0.38)	−1.33 (−1.4)
Musculoskeletal and connective tissue disorders	2,341	0.83 (0.79–0.86)	0.83 (81.44)	0.83 (0.81)	−0.26 (−0.32)
Injury, poisoning and procedural complications	4,771	0.75 (0.73–0.77)	0.77 (371.3)	0.77 (0.75)	−0.38 (−0.42)
Psychiatric disorders	803	0.26 (0.25–0.28)	0.27 (1623.52)	0.28 (0.26)	−1.86 (−1.96)
Social circumstances	85	0.34 (0.28–0.43)	0.35 (105.63)	0.35 (0.29)	−1.53(−1.84)
Eye disorders	462	0.42 (0.38–0.46)	0.43 (363.42)	0.43 (0.4)	−1.23(−1.36)
Hepatobiliary disorders	178	0.39 (0.34–0.45)	0.39 (169.36)	0.39 (0.35)	−1.35(−1.57)
Metabolism and nutrition disorders	326	0.29 (0.26–0.32)	0.29 (562.35)	0.29 (0.27)	−1.76(−1.92)
Cardiac disorders	437	0.38 (0.35–0.42)	0.39 (430.78)	0.39 (0.36)	−1.36 (−1.5)
Vascular disorders	382	0.36 (0.32–0.4)	0.36 (434.77)	0.36 (0.33)	−1.46(−1.61)
Product issues	303	0.3 (0.27–0.34)	0.3 (489.36)	0.31 (0.28)	−1.71(−1.88)
Surgical and medical procedures*	1,834	2.39 (2.28–2.5)	2.34 (1424.74)	2.34 (2.25)	1.22 (1.16)
Renal and urinary disorders	342	0.31 (0.28–0.35)	0.32 (510.66)	0.32 (0.29)	−1.65(−1.81)
Reproductive system and breast disorders	128	0.33 (0.28–0.39)	0.33 (175.04)	0.33 (0.29)	−1.6 (−1.85)
Neoplasms benign, malignant and unspecified (incl cysts and polyps)	753	0.44 (0.41–0.47)	0.45 (531.47)	0.45 (0.42)	−1.16(−1.27)
Ear and labyrinth disorders	184	0.77 (0.67–0.89)	0.77 (12.6)	0.77 (0.68)	−0.38(−0.59)
Endocrine disorders	25	0.17 (0.12–0.26)	0.17 (98.06)	0.17 (0.13)	−2.52(−3.09)
Congenital, familial and genetic disorders	14	0.09 (0.06–0.16)	0.09 (122.33)	0.09 (0.06)	−3.41(−4.15)
Pregnancy, puerperium and perinatal conditions	48	0.23 (0.17–0.3)	0.23 (126.78)	0.23 (0.18)	−2.14 (−2.55)

Asterisks (*) indicate statistically significant signals in algorithm; ROR, reporting odds ratio; PRR, proportional reporting ratio; EBGM, empirical Bayesian geometric mean; EBGM05, the lower limit of the 95% CI of EBGM; IC, information component; IC025, the lower limit of the 95% CI of the IC; CI, confidence interval; AEs, adverse events.

### 3.3 Distribution of AEs at the PT level

We further analyzed all signals at the PT level, focusing on PTs with a count of ≥45 with the highest signal strength and the top 70 most frequent PTs detected in both the FAERS and VigiAccess databases, as listed in Table 3 and Supplementary Table 4.

**Table 3:** Signal strength of reports of IXE with preferred terms (PTs) ≥ 45 from FAERS.

PT	Case numbers	ROR(95%Cl)	PRR(χ^2^)	EBGM(EBGM05)	IC(IC025)
Injection site pain	3,581	16.05(15.51–16.61)	15.06 (46163.17)	14.75 (14.33)	3.88 (3.83)
Injection site erythema	2,014	26.19(25.03–27.41)	25.26 (45249.64)	24.36 (23.45)	4.61 (4.54)
Injection site swelling	1,519	28.29 (26.86–29.8)	27.53 (37306.84)	26.46 (25.33)	4.73 (4.65)
Injection site reaction	1,459	30.4 (28.83–32.06)	29.62 (38626.91)	28.37 (27.14)	4.83 (4.75)
Injection site pruritus	695	14.49(13.44–15.63)	14.32 (8434.19)	14.03 (13.17)	3.81 (3.7)
Injection site urticaria	606	32.49(29.93–35.27)	32.14 (17432.15)	30.68 (28.64)	4.94 (4.82)
Injection site mass	549	15.18(13.94–16.52)	15.03 (7034.93)	14.72 (13.71)	3.88 (3.75)
Injection site hemorrhage	512	7.79 (7.13–8.5)	7.72 (2964.55)	7.64 (7.1)	2.93 (2.81)
Nasopharyngitis	475	2.81 (2.56–3.07)	2.79 (545.06)	2.78 (2.58)	1.48 (1.34)
Injection site rash	468	20.43(18.63–22.41)	20.27 (8317.37)	19.69 (18.22)	4.3 (4.16)
Injection site warmth	451	38.5 (35–42.35)	38.19 (15,434)	36.13 (33.36)	5.18 (5.04)
Urticaria	436	3.12 (2.84–3.43)	3.1 (618.94)	3.09 (2.86)	1.63 (1.49)
Injection site bruising	415	6.66 (6.05–7.34)	6.62 (1962.04)	6.56 (6.05)	2.71 (2.57)
Sinusitis	397	4.34 (3.93–4.79)	4.31 (1005.2)	4.29 (3.95)	2.1 (1.96)
Influenza	293	2.81 (2.51–3.16)	2.81 (339.55)	2.8 (2.54)	1.48 (1.32)
Cellulitis	261	6.08 (5.38–6.87)	6.06 (1093.16)	6.01 (5.43)	2.59 (2.41)
Ear infection	213	8.66 (7.56–9.91)	8.63 (1417.93)	8.53 (7.61)	3.09 (2.89)
Upper respiratory tract infection	185	4.51 (3.9–5.21)	4.5 (500.14)	4.47 (3.96)	2.16 (1.95)
Bronchitis	175	2.76 (2.38–3.21)	2.76 (195.63)	2.75 (2.43)	1.46 (1.24)
Fungal infection	172	5.84 (5.02–6.78)	5.82 (681.08)	5.78 (5.1)	2.53 (2.31)
Herpes zoster	135	2.53 (2.14–3)	2.53 (124.68)	2.53 (2.19)	1.34 (1.09)
Oral candidiasis	134	12.94 (10.9–15.35)	12.91 (1443.68)	12.68 (10.98)	3.66 (3.41)
Candida infection	125	7.24 (6.07–8.63)	7.22 (663.12)	7.16 (6.17)	2.84 (2.58)
Pharyngitis streptococcal	111	11.46 (9.49–13.82)	11.43 (1038.89)	11.25 (9.62)	3.49 (3.22)
Inflammatory bowel disease	98	20.41(16.69–24.97)	20.38 (1751.5)	19.79 (16.73)	4.31 (4.01)
Colitis	94	2.72 (2.22–3.33)	2.72 (101.63)	2.71 (2.29)	1.44 (1.14)
Staphylococcal infection	93	3.83 (3.12–4.69)	3.82 (192.71)	3.81 (3.21)	1.93 (1.63)
Irritable bowel syndrome	83	4.65 (3.74–5.77)	4.64 (235.56)	4.62 (3.85)	2.21 (1.89)
Tooth infection	79	6.62 (5.3–8.26)	6.61 (372.37)	6.55 (5.44)	2.71 (2.39)
Respiratory tract infection	66	2.74 (2.15–3.49)	2.74 (72.59)	2.73 (2.23)	1.45 (1.1)
Diverticulitis	64	2.54 (1.99–3.25)	2.54 (59.41)	2.53 (2.06)	1.34 (0.98)
Immune system disorder	60	4.84 (3.75–6.24)	4.83 (181.03)	4.8 (3.88)	2.26 (1.89)
Kidney infection	53	2.87 (2.19–3.76)	2.87 (64.13)	2.86 (2.28)	1.51 (1.12)
Conjunctivitis	52	2.95 (2.25–3.88)	2.95 (66.69)	2.94 (2.34)	1.56 (1.16)
Tuberculosis	52	4.54 (3.45–5.96)	4.54 (142.36)	4.51 (3.59)	2.17 (1.78)
Gastroenteritis viral	47	2.95 (2.22–3.93)	2.95 (60.38)	2.94 (2.31)	1.56 (1.14)
Malignant melanoma	46	3.55 (2.66–4.74)	3.55 (83.71)	3.53 (2.77)	1.82 (1.4)

In the FAERS database, a total of 192 positive signals were identified that met the criteria for positivity across all four algorithms. Among them, injection site warmth and injection site urticaria have the strongest signals. The top 70 positive signals of IXE are listed in Supplementary Table 5. In our data analysis, injection site reactions, hypersensitivity reactions, fungal infections, upper respiratory tract infections, and inflammatory bowel disease were consistent with the instructions and medication warnings. In addition, unexpected AEs not listed on the label have been identified, such as cellulitis, ear infection, bronchitis, herpes zoster, tooth infection, diverticulitis, kidney infection, gastroenteritis viral and malignant melanoma. In the VigiAccess database, injection site pain (7.17%), injection site erythema (4.19%), and injection site swelling (2.93%) were the three most frequently reported AEs. The ranking of PTs differed slightly between the FAERS and Vigiaccess databases, but the overall reported PTs were similar.

### 3.4 Subgroup analysis

Further subgroup analysis was conducted on the 50 most common AEs associated with IXE that met the criteria for all four algorithms. AEs occurring exclusively in males were serum sickness, aphthous ulcer, necrotising fasciitis, miliaria, and large intestine perforation while those occurring exclusively in females included immune system disorder. The specific details are provided in Supplementary Tables 6, 7. In adolescent patients under the age of 18, common AEs in addition to those mentioned on the drug label include alopecia, drug hypersensitivity, and vitiligo. In the patient population aged 18–64 years, additional AEs identified included herpes zoster, Spinal operation, immune system disorder, antibiotic therapy, cardiac surgery, and multiple allergies. For patients over the age of 64, common AEs included coronary arterial stent insertion, and bursitis (Supplementary Tables 8–10).

### 3.5 Sensitivity analysis

IXE is commonly used in combination with other medications such as clobetasol, prednisone, triamcinolone, betamethasone calcipotriene, and vitamin D3. After excluding reports of concomitant use of other therapeutic agents, we identified 28,633 reports involving 53,765 AEs. Cellulitis, herpes zoster and malignant melanoma were identified as unexpected AEs (Supplementary Table 11).

### 3.6 Onset time of events

**Figure 3:**
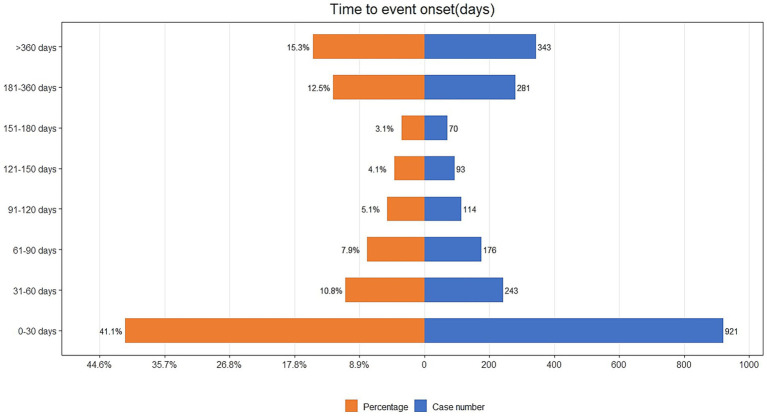
Time to onset of IXE-related AEs.

**Figure 4:**
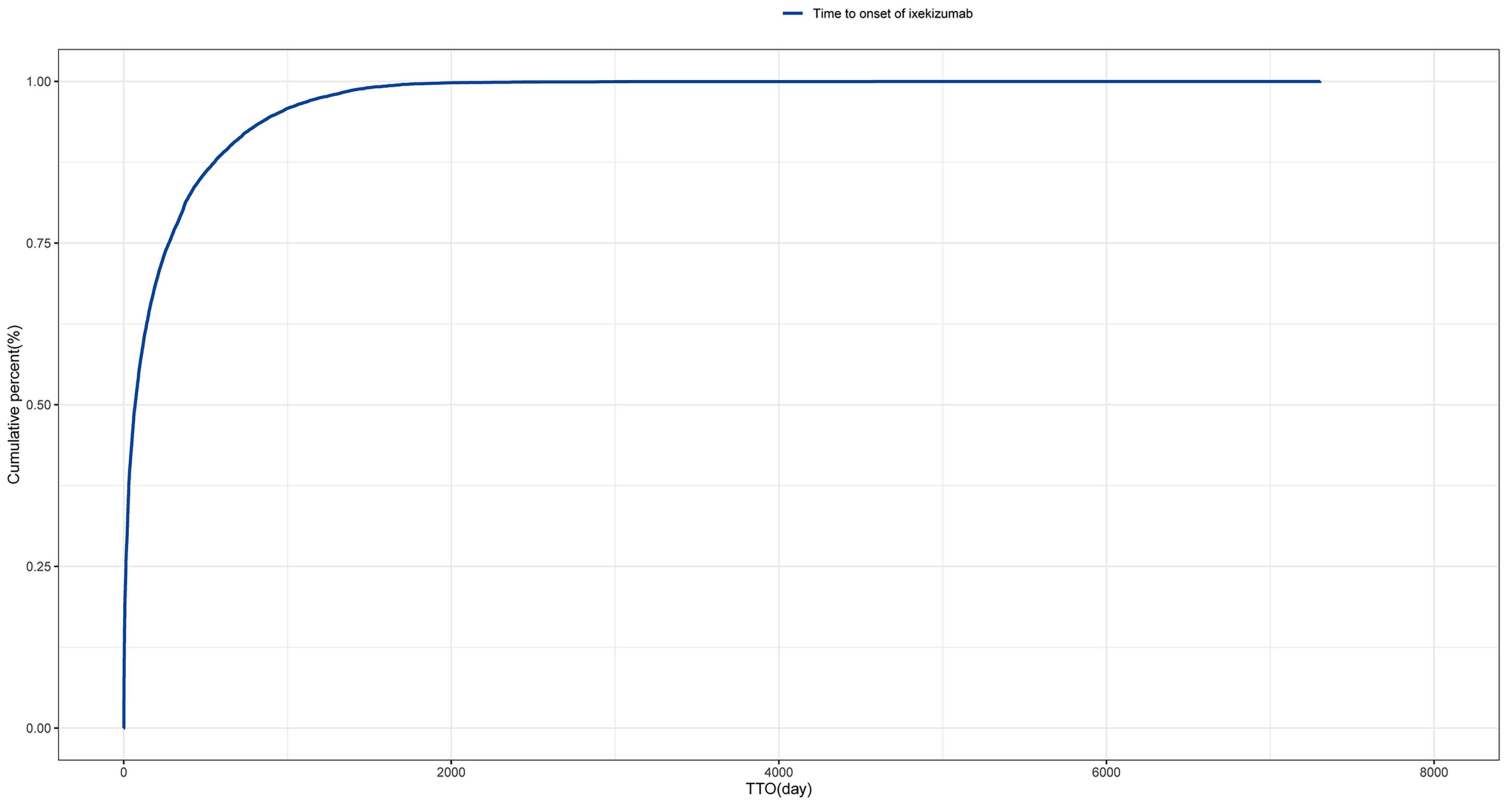
Cumulative incidence of adverse events related to tralokinumab over time.

The time-to-event onset represents the time interval between the date of drug administration and the occurrence of the AE. As shown in Figure 3, the majority of AEs associated with IXE occurred primarily within the first month of treatment. Additionally, the cumulative incidence curve of AEs is illustrated in Figure 4. The Weibull distribution analysis exhibits a shape parameter less than 1, indicating a higher probability of failure in the early stage, referred to as the early failure mode. Table 4 provides the detailed parameters of this analysis.

**Table 4:** Time to onset of ixekizumab-associated adverse events and Weibull distribution analysis.

Drug	TTO(days)		Weibull distribution		
	Case reports	Median(d)(IQR)	Scale parameter: α(95%CI)	Shape parameter: β(95%CI)	Type
Ixekizumab	28,889	70(14,276)	143.6(140.0,147.2)	0.60(0.59,0.61)	Early failure

TTO, time to onset; CI, confidence interval; IQR, interquartile range.

## 4 Discussion

Due to the chronic nature of psoriasis, long-term treatment and continuous safety monitoring are essential. This study represents the first extensive and systematic pharmacovigilance analysis of AEs associated with IXE using the FAERS and VigiAccess databases following its market approval. By comparing and analyzing IXE-related adverse events reported in the VigiAccess and FAERS databases, this study provides an in-depth understanding of the common, novel, and rare AEs associated with this drug. Through data mining, we confirmed previously identified AEs listed on the drug labeling, such as injection site reactions, nasopharyngitis, infections, urticaria, and inflammatory bowel disease. Additionally, we identified unexpected AEs, including cellulitis, bronchitis, and herpes zoster.

Multiple clinical trials have demonstrated that the common AEs associated with IXE include nasopharyngitis, upper respiratory tract infections, and injection-site reactions. Other AEs include bronchitis, sinusitis, inflammatory bowel disease, among others (18, 19). These findings are consistent with our conclusions.

In our study, injection site reactions were the most commonly observed AEs. Research indicates that injection-site reactions are associated with the components of IXE. The new citrate-free formulation was developed to enhance the overall patient experience, indicating a lower reported frequency of injection site reactions and reduced injection site pain, thereby enhancing tolerability and patient satisfaction (20).

Another notable AEs is infection. Previously, concerns have been raised regarding the safety profile of immunomodulators, primarily due to the potential for these agents to increase the risk of infections (21, 22). The infection issues caused by biological agents, represented by IXE, also deserve attention. IL-17 is involved in mucocutaneous defense. Inhibition of IL-17 may compromise the immune system and increase the risk of infections, particularly enhancing susceptibility to staphylococcal infections and mucocutaneous candidiasis (23). Across multiple clinical trials, the most common infections reported among all AEs were nasopharyngitis, upper respiratory tract infections and bronchitis. Infections were reported among 4,307 patients with PSO (62.5%, IR 23.9 per 100 PY), 759 patients with PSA (54.2%, IR 33.8 per 100 PY) (17, 23). The study also reported that the opportunistic infections observed after the use of IXE were primarily oral candidiasis, esophageal candidiasis, and oral fungal infections (18, 19, 24, 25). Our study indicates that the most commonly reported infections include nasopharyngitis and sinusitis. Additionally, influenza, cellulitis, ear infection, upper respiratory tract infection, bronchitis, fungal infection, oral candidiasis, and candida infection also appeared among the positive signals. Severe infections, such as mucosal candidiasis, often necessitate the discontinuation of IXE and prolonged systemic antimicrobial therapy, which may lead to the exacerbation of the underlying condition and an increased disease burden. Therefore, strictly monitoring and preventing the occurrence of common AEs during the treatment process is crucial. This approach can effectively prevent the progression of serious AEs, thereby improving drug retention and patient adherence.

Our study identified a positive signal for inflammatory bowel disease (IBD). IBD, which includes ulcerative colitis (UC) and Crohn’s disease (CD), is primarily characterized by inflammation of the gastrointestinal tract, weight loss, and diarrhea. Although UC and Crohn’s disease CD do not manifest identically, both conditions have significant long-term impacts on patients.

At present, this condition is incurable and necessitates lifelong treatment to manage symptoms, prevent complications, and ultimately enhance the quality of life (26). The Phase 3 trials UNCOVER-2 and UNCOVER-3 explored the efficacy and safety of IXE in patients with moderate-to-severe psoriasis. In these studies, 4 cases of CD and 7 cases of ulcerative colitis UC were reported in the IXE treatment groups, compared to zero cases in the placebo groups (27). In 2023, Deng Z et al. published a comprehensive paper on the prevalence, clinical characteristics, and management of IBD events associated with anti-IL-17 therapies. Their study also involved a retrospective analysis of case reports and case series of anti-IL-17 drug-induced IBD cases from 2015 to 2022. A total of 388 cases of gastrointestinal inflammatory events associated with IL-17 inhibitors were reported, including 268 cases of IBD induced by secukinumab and IXE. The main presenting symptoms were diarrhea (90.9%), abdominal pain (57.6%), and bloody diarrhea (51.5%), with a total of 120 cases diagnosed as colitis (28). Furthermore, multiple case reports have highlighted the development of steroid-refractory ulcerative colitis with a superimposed cytomegalovirus infection, Crohn’s-like colitis, CD, and other gastrointestinal disorders in patients treated with IXE (29–32).

In a nationwide Danish cohort study, nearly one-quarter of IBD patients with psoriasis had a family history of psoriasis. Interestingly, the risk of developing IBD—including Crohn’s disease and ulcerative colitis—increases with the severity of psoriasis (33, 34). Limited data suggest an association between IL-17 inhibitor therapy and the development of IBD.

Researchers have proposed a gut-skin-joint axis, which illustrates how the gut microbiota, disturbances in immune balance, and increased gut permeability can influence the skin and joints, affecting their homeostasis and leading to inflammation (35). The IL-23/Th17 axis plays a critical role in the pathogenesis of both diseases. Although IL-23 inhibition has been proven to be an effective mechanism in the treatment of IBD in clinical trials, there is no evidence that IL-17 inhibition provides any benefits; instead, it is associated with an increase in AEs. Researchers hypothesize that these results may reflect the heterogeneous functions of various IL-17 subtypes. Some of these subtypes may act as pro-inflammatory mediators, while others appear to have a protective role against IBD (36, 37).

Studies have indicated that Th17 cells infiltrate the intestinal mucosa of IBD patients more extensively than in healthy controls. Furthermore, the levels of IL-17, a cytokine secreted by Th17 cells, are elevated in these patients (38). Clinical trials utilizing IL-17 inhibitors for the treatment of IBD have not shown any therapeutic benefits, and in certain cases, have been associated with disease worsening. This indicates that while IL-17 serves a pro-inflammatory function in psoriasis, it may play a protective role in maintaining gut integrity in IBD (39, 40).

However, disproportionality analysis only suggests a statistical association, not causality. The causal relationship among psoriasis, IL-17 inhibitors, and IBD remains incompletely understood. Future research should prioritize elucidating the potential pathophysiological mechanisms linking psoriasis and IL-17 blockade to the development of IBD, rather than focusing primarily on statistical associations.

In addition, our study revealed several unexpected AEs. Notably, our study also identified herpes zoster as an unexpected AE. Herpes zoster is a disease caused by the reactivation of the Varicella-Zoster, which can lead to severe pain, rash, and postherpetic neuralgia, affecting sleep, mood, and daily activities, significantly reducing the quality of life (41). In the COAST-W study, one case of herpes zoster was reported in the group receiving IXE every 4 weeks (42). Additionally, two cases of herpes zoster were reported in the same dosing group in the COAST-X study. The study found that herpes zoster is one of the most common opportunistic infections associated with IXE treatment. Herpes zoster was reported among 120 patients with PSO (1.7%, IR 0.6 per 100 PY), 16 patients with PSA (1.1%, IR 0.7 per 100 PY) (43). The exact mechanism by which IXE leads to herpes zoster remains to be elucidated. During IXE treatment, preventive measures, including vaccination, should be implemented for individuals with compromised immune status. Close monitoring and follow-up are crucial for further reducing this risk.

In our analysis, an unexpected condition showed a positive signal—malignant melanoma—which warrants clinical attention. Cutaneous malignancies are a significant concern for patients with psoriasis due to their association with sun exposure (44). Malignant melanoma results from the malignant transformation of melanocytes, which are melanin-producing cells derived from the neural crest. It is characterized by local invasion, recurrence, early metastasis, drug resistance, and high mortality rates (45). Immune checkpoint inhibitors ICIs, particularly those targeting programmed cell death 1 (PD-1), have significantly transformed the landscape of cancer treatment.

A retrospective study including a total of 61,692 patients with psoriasis compared the incidence of malignant melanoma with that in a matched control group, showing that patients with psoriasis, particularly those with severe disease, had a higher risk of developing malignant melanoma, especially melanoma *in situ* (46). In contrast, other studies have reported similar incidence rates of these tumors compared to the general population. Additional evidence suggests that these patients might have a higher risk of cutaneous malignancies, especially for non-melanoma skin cancer, compared with psoriasis-free patients., relative to individuals without psoriasis (47). In addition, evidence has shown that psoralen plus ultraviolet A therapy is associated with an increased risk of non-melanoma skin cancer, but not with melanoma (48). Limited studies suggest that patients treated with methotrexate or tumor necrosis factor-alpha inhibitors may have a slightly increased risk of melanoma (49, 50). In contrast, studies have evaluated the risk of developing melanoma with conventional therapies and newer agents for the treatment of psoriasis. No significant increase in the risk of melanoma was found associated with the use of anti-psoriatic treatments (51). Shamarke et al. reported an increased risk of melanoma in patients receiving biologic agents compared to those receiving conventional systemic therapies, although the difference was not statistically significant (44). The development and progression of melanoma is a highly complex process, and the chronic inflammatory state associated with long-standing psoriasis, as well as its various treatment modalities, may exert differential influences on the development and progression of melanoma.

Currently, there is limited real-world evidence regarding the association between IL-17 inhibitors and malignant melanoma. A 2024 systematic review assessed melanoma and non-melanoma skin cancer risks in psoriasis and psoriatic arthritis patients on targeted therapies. The pooled melanoma incidence rates were: 0.06 (95% CI, 0.02–0.18) per 100 patient-years for IL-17 inhibitors, 0.10 (95% CI, 0.05–0.21) for IL-23 inhibitors, and 0.09 (95% CI, 0.03–0.28) for JAK inhibitors (52). Although the pathophysiological mechanisms by which IXE contributes to melanoma development remain unclear, studies in melanoma animal models have demonstrated that IL-17A-deficient mice are susceptible to developing spontaneous melanoma (53). Moreover, studies have shown that increased IL-17 signaling and elevated serum IL-17 levels are positively correlated with better therapeutic responses in melanoma patients treated with dual cytotoxic T-lymphocyte antigen 4 and PD-1 immune checkpoint inhibitors, and are linked to prolonged overall survival for these patients (54). These findings suggest a potential association between IL-17 and melanoma, but cannot be directly attributed to the effect of IL-17 inhibition. The development and progression of melanoma is a highly complex process likely influenced by the interplay of multiple factors. Further research is needed to better understand the potential link between IXE treatment for psoriasis and melanoma, particularly long-term observational studies with matched control groups and adjustment for known risk factors. Such studies will be essential to elucidate the true nature of this potential association.

Subgroup analysis revealed that and serum sickness in males require additional attention, while females should be vigilant against the risks of immune system disorders. For patients under 18 years of age, it is important to monitor for alopecia and vitiligo. For patients aged 18 to 65, attention should be given to immune-related issues such as herpes zoster. Addressing these concerns can help improve medication adherence.

In addition, sensitivity analysis revealed persistent unexpected AEs associated with IXE monotherapy, such as cellulitis, herpes zoster and malignant melanoma. These AEs may exert negative effects on treatment satisfaction, adherence, and efficacy.

This study also performed a temporal analysis of AEs and utilized the Weibull distribution to model and predict the timing of these events. This approach provides a robust temporal framework for monitoring drug-related AEs, enabling more informed and timely clinical interventions. The results highlight the importance of monitoring for specific AEs, especially during the first month of IXE treatment. Additionally, a gradual reduction in AEs was observed as time progressed. This underscores the importance of closely monitoring AEs during the initial phase of drug administration while also maintaining continuous attention to the long-term safety profile of the medication. Monitoring and preventing at specific stages are crucial for managing unexpected AEs, which helps to improve the satisfaction, efficacy, and safety of medication use.

This study has some limitations. First, due to the spontaneous reporting nature of the FAERS and VigiAccess databases, they may inherently contain missing or inaccurate data. Additionally, there is a potential risk of over-reporting for serious adverse events, which may be more likely to be reported due to frequent follow-up visits or medical consultations. Second, while the sensitivity analysis partially accounted for the influence of certain medications on the results, other confounding factors may still exist due to the nature of real-world data. Therefore, the results should be interpreted with caution. Finally, although disproportionality analysis effectively identified positive signals for AEs and detected unexpected AEs, it is not capable of establishing a causal relationship between the events. Therefore, further prospective studies are essential to validate the unexpected AEs identified in this study.

## 5 Conclusion

This study explored the overall real-world safety profile of IXE. Our analysis confirmed several known adverse events. Additionally, several potential unexpected adverse events were identified, including herpes zoster, and cellulitis. Furthermore, this study highlights the importance of monitoring during the first month following IXE initiation. Prospective studies are warranted to validate the findings of this study.

## Data Availability

The original contributions presented in the study are included in the article/Supplementary material, further inquiries can be directed to the corresponding authors.
