# Supplementary material for: Real-world safety of ixekizumab: a disproportionality analysis using the FDA adverse event reporting system and the VigiAccess databases
Source: Front Med (Lausanne). 2025 Oct 13;12:1652401. doi: 10.3389/fmed.2025.1652401 (PMC12554608; doi:10.3389/fmed.2025.1652401)
Supplement: Supplementary file 1 [file Data_Sheet_1.docx]

Supplementary Material

# Supplementary Tables

Supplementary Table 1:

Two-by-two contingency table for disproportionality analyses

|  | Target AEs | Other AEs | Total |
| --- | --- | --- | --- |
|  | a | b | a+b |
| Other drugs | c | d | c+d |
| Total | a+c | b+d | a+b+c+d |

Abbreviation: AEs, adverse events; a, number of reports containing both the target drug and target adverse drug reaction; b, number of reports containing other adverse drug reaction of the target drug; c, number of reports containing the target adverse drug reaction of other drugs; d, number of reports containing other drugs and other adverse drug reactions.

Supplementary Table 2:

Four major algorithms used for signal detection

| Algorithms | Equation | Criteria |
| --- | --- | --- |
| ROR | ROR=ad/b/c | lower limit of 95% CI>1, N≥3 |
|  | 95%CI=e^ln(ROR)±1.96(1/a+1/b+1/c+1/d)^0.5^ |  |
| PRR | PRR=a(c+d)/c/(a+b) | PRR≥2, χ^2^≥4, N≥3 |
|  | χ^2^=[(ad-bc)^2](a+b+c+d)/[(a+b)(c+d)(a+c)(b+d)] |  |
| BCPNN | IC=log_2_a(a+b+c+d)(a+c)(a+b) | IC025>0 |
|  | 95%CI= E(IC) ± 2V(IC)^0.5 |  |
| MGPS | EBGM=a(a+b+c+d)/(a+c)/(a+b) | EBGM05>2 |
|  | 95%CI=e^ln(EBGM)±1.96(1/a+1/b+1/c+1/d)^0.5^ |  |

Abbreviation: a, number of reports containing both the target drug and target adverse drug reaction; b, number of reports containing other adverse drug reaction of the target drug; c, number of reports containing the target adverse drug reaction of other drugs; d, number of reports containing other drugs and other adverse drug reactions. 95%CI, 95% confidence interval; N, the number of reports; χ2, chi-squared; IC, information component; IC025, the lower limit of 95% CI of the IC; E(IC), the IC expectations; V(IC), the variance of IC; EBGM, empirical Bayesian geometric mean; EBGM05, the lower limit of 95% CI of EBGM.

Supplementary Table 3:

Distribution of AE Signals by System Organ Class (SOC) in the VigiAccess Database

| SOC | Case number | Report rate |
| --- | --- | --- |
| General disorders and administration site conditions | 20797 | 31.02% |
| Infections and infestations | 8989 | 13.41% |
| Skin and subcutaneous tissue disorders | 8863 | 13.22% |
| Injury, poisoning and procedural complications | 5996 | 8.94% |
| Gastrointestinal disorders | 3957 | 5.90% |
| Musculoskeletal and connective tissue disorders | 3521 | 5.25% |
| Nervous system disorders | 2293 | 3.42% |
| Respiratory, thoracic and mediastinal disorders | 1857 | 2.77% |
| Investigations | 1819 | 2.71% |
| Surgical and medical procedures | 1728 | 2.58% |
| Immune system disorders | 1179 | 1.76% |
| Psychiatric disorders | 1132 | 1.69% |
| Neoplasms benign, malignant and unspecified | 822 | 1.23% |
| Eye disorders | 664 | 0.99% |
| Vascular disorders | 552 | 0.82% |
| Cardiac disorders | 507 | 0.76% |
| Metabolism and nutrition disorders | 499 | 0.74% |
| Renal and urinary disorders | 420 | 0.63% |
| Blood and lymphatic system disorders | 312 | 0.47% |
| Ear and labyrinth disorders | 273 | 0.41% |
| Hepatobiliary disorders | 230 | 0.34% |
| Reproductive system and breast disorders | 228 | 0.34% |
| Product issues | 214 | 0.32% |
| Social circumstances | 76 | 0.11% |
| Pregnancy, puerperium and perinatal conditions | 55 | 0.08% |
| Endocrine disorders | 48 | 0.07% |
| Congenital, familial and genetic disorders | 20 | 0.03% |

Supplementary Table 4:

Top 70 AEs with the highest percentage of signal detection in the VigiAccess database

| PT | Case number | Report rate |
| --- | --- | --- |
| Injection site pain | 6197 | 7.17% |
| Injection site erythema | 3623 | 4.19% |
| Injection site swelling | 2533 | 2.93% |
| Injection site reaction | 2447 | 2.83% |
| Injection site pruritus | 1278 | 1.48% |
| Pruritus | 1058 | 1.22% |
| Nasopharyngitis | 990 | 1.15% |
| Arthralgia | 934 | 1.08% |
| Diarrhoea | 864 | 1.00% |
| Injection site mass | 851 | 0.99% |
| Fatigue | 822 | 0.95% |
| Injection site urticaria | 807 | 0.93% |
| Pain | 739 | 0.86% |
| Injection site rash | 715 | 0.83% |
| Nausea | 702 | 0.81% |
| Injection site warmth | 699 | 0.81% |
| Injection site haemorrhage | 660 | 0.76% |
| Headache | 640 | 0.74% |
| Urticaria | 633 | 0.73% |
| Injection site bruising | 611 | 0.71% |
| Influenza | 527 | 0.61% |
| Pyrexia | 499 | 0.58% |
| Infection | 498 | 0.58% |
| Hypersensitivity | 494 | 0.57% |
| Erythema | 481 | 0.56% |
| Sinusitis | 480 | 0.56% |
| Urinary tract infection | 460 | 0.53% |
| Illness | 432 | 0.50% |
| Pain in extremity | 407 | 0.47% |
| Oropharyngeal pain | 396 | 0.46% |
| Cough | 375 | 0.43% |
| Injection site induration | 356 | 0.41% |
| Alopecia | 349 | 0.40% |
| Pneumonia | 333 | 0.39% |
| Dizziness | 329 | 0.38% |
| Oral candidiasis | 309 | 0.36% |
| Dyspnoea | 298 | 0.34% |
| Peripheral swelling | 297 | 0.34% |
| Eczema | 291 | 0.34% |
| Ear infection | 283 | 0.33% |
| Death | 272 | 0.31% |
| Injection site inflammation | 262 | 0.30% |
| Cellulitis | 262 | 0.30% |
| Drug hypersensitivity | 259 | 0.30% |
| Upper respiratory tract infection | 255 | 0.30% |
| Bronchitis | 242 | 0.28% |
| Fungal infection | 239 | 0.28% |
| Influenza like illness | 236 | 0.27% |
| Back pain | 235 | 0.27% |
| Candida infection | 233 | 0.27% |
| Abdominal pain | 229 | 0.27% |
| Vomiting | 227 | 0.26% |
| Asthenia | 213 | 0.25% |
| Weight increased | 210 | 0.24% |
| Fall | 208 | 0.24% |
| Weight decreased | 205 | 0.24% |
| Injection site discomfort | 203 | 0.24% |
| Abdominal pain upper | 200 | 0.23% |
| Herpes zoster | 198 | 0.23% |
| Feeling abnormal | 196 | 0.23% |
| Abdominal discomfort | 189 | 0.22% |
| Swelling | 179 | 0.21% |
| Injection site oedema | 177 | 0.20% |
| Crohn's disease | 176 | 0.20% |
| Gastrointestinal disorder | 175 | 0.20% |
| Stress | 171 | 0.20% |
| Injection site hypersensitivity | 168 | 0.19% |
| Joint swelling | 166 | 0.19% |
| Gait disturbance | 164 | 0.19% |
| Colitis ulcerative | 162 | 0.19% |

Supplementary Table 5:

Top 70 adverse events meeting the positive signal threshold at the PT level from FAERS data

| PT | Case numbers | ROR(95%CI) | PRR(χ2) | EBGM(EBGM05) | IC(IC025) |
| --- | --- | --- | --- | --- | --- |
| Injection site pain | 3,581 | 16.05 ( 15.51 - 16.61 ) | 15.06 ( 46163.17 ) | 14.75 ( 14.33 ) | 3.88 ( 3.83 ) |
| Injection site erythema | 2,014 | 26.19 ( 25.03 - 27.41 ) | 25.26 ( 45249.64 ) | 24.36 ( 23.45 ) | 4.61 ( 4.54 ) |
| Injection site swelling | 1,519 | 28.29 ( 26.86 - 29.8 ) | 27.53 ( 37306.84 ) | 26.46 ( 25.33 ) | 4.73 ( 4.65 ) |
| Injection site reaction | 1,459 | 30.4 ( 28.83 - 32.06 ) | 29.62 ( 38626.91 ) | 28.37 ( 27.14 ) | 4.83 ( 4.75 ) |
| Injection site pruritus | 695 | 14.49 ( 13.44 - 15.63 ) | 14.32 ( 8434.19 ) | 14.03 ( 13.17 ) | 3.81 ( 3.7 ) |
| Injection site urticaria | 606 | 32.49 ( 29.93 - 35.27 ) | 32.14 ( 17432.15 ) | 30.68 ( 28.64 ) | 4.94 ( 4.82 ) |
| Injection site mass | 549 | 15.18 ( 13.94 - 16.52 ) | 15.03 ( 7034.93 ) | 14.72 ( 13.71 ) | 3.88 ( 3.75 ) |
| Injection site haemorrhage | 512 | 7.79 ( 7.13 - 8.5 ) | 7.72 ( 2964.55 ) | 7.64 ( 7.1 ) | 2.93 ( 2.81 ) |
| Nasopharyngitis | 475 | 2.81 ( 2.56 - 3.07 ) | 2.79 ( 545.06 ) | 2.78 ( 2.58 ) | 1.48 ( 1.34 ) |
| Injection site rash | 468 | 20.43 ( 18.63 - 22.41 ) | 20.27 ( 8317.37 ) | 19.69 ( 18.22 ) | 4.3 ( 4.16 ) |
| Injection site warmth | 451 | 38.5 ( 35 - 42.35 ) | 38.19 ( 15434 ) | 36.13 ( 33.36 ) | 5.18 ( 5.04 ) |
| Urticaria | 436 | 3.12 ( 2.84 - 3.43 ) | 3.1 ( 618.94 ) | 3.09 ( 2.86 ) | 1.63 ( 1.49 ) |
| Injection site bruising | 415 | 6.66 ( 6.05 - 7.34 ) | 6.62 ( 1962.04 ) | 6.56 ( 6.05 ) | 2.71 ( 2.57 ) |
| Sinusitis | 397 | 4.34 ( 3.93 - 4.79 ) | 4.31 ( 1005.2 ) | 4.29 ( 3.95 ) | 2.1 ( 1.96 ) |
| Influenza | 293 | 2.81 ( 2.51 - 3.16 ) | 2.81 ( 339.55 ) | 2.8 ( 2.54 ) | 1.48 ( 1.32 ) |
| Cellulitis | 261 | 6.08 ( 5.38 - 6.87 ) | 6.06 ( 1093.16 ) | 6.01 ( 5.43 ) | 2.59 ( 2.41 ) |
| Ear infection | 213 | 8.66 ( 7.56 - 9.91 ) | 8.63 ( 1417.93 ) | 8.53 ( 7.61 ) | 3.09 ( 2.89 ) |
| Upper respiratory tract infection | 185 | 4.51 ( 3.9 - 5.21 ) | 4.5 ( 500.14 ) | 4.47 ( 3.96 ) | 2.16 ( 1.95 ) |
| Bronchitis | 175 | 2.76 ( 2.38 - 3.21 ) | 2.76 ( 195.63 ) | 2.75 ( 2.43 ) | 1.46 ( 1.24 ) |
| Fungal infection | 172 | 5.84 ( 5.02 - 6.78 ) | 5.82 ( 681.08 ) | 5.78 ( 5.1 ) | 2.53 ( 2.31 ) |
| Herpes zoster | 135 | 2.53 ( 2.14 - 3 ) | 2.53 ( 124.68 ) | 2.53 ( 2.19 ) | 1.34 ( 1.09 ) |
| Oral candidiasis | 134 | 12.94 ( 10.9 - 15.35 ) | 12.91 ( 1443.68 ) | 12.68 ( 10.98 ) | 3.66 ( 3.41 ) |
| Candida infection | 125 | 7.24 ( 6.07 - 8.63 ) | 7.22 ( 663.12 ) | 7.16 ( 6.17 ) | 2.84 ( 2.58 ) |
| Pharyngitis streptococcal | 111 | 11.46 ( 9.49 - 13.82 ) | 11.43 ( 1038.89 ) | 11.25 ( 9.62 ) | 3.49 ( 3.22 ) |
| Inflammatory bowel disease | 98 | 20.41 ( 16.69 - 24.97 ) | 20.38 ( 1751.5 ) | 19.79 ( 16.73 ) | 4.31 ( 4.01 ) |
| Colitis | 94 | 2.72 ( 2.22 - 3.33 ) | 2.72 ( 101.63 ) | 2.71 ( 2.29 ) | 1.44 ( 1.14 ) |
| Staphylococcal infection | 93 | 3.83 ( 3.12 - 4.69 ) | 3.82 ( 192.71 ) | 3.81 ( 3.21 ) | 1.93 ( 1.63 ) |
| Irritable bowel syndrome | 83 | 4.65 ( 3.74 - 5.77 ) | 4.64 ( 235.56 ) | 4.62 ( 3.85 ) | 2.21 ( 1.89 ) |
| Tooth infection | 79 | 6.62 ( 5.3 - 8.26 ) | 6.61 ( 372.37 ) | 6.55 ( 5.44 ) | 2.71 ( 2.39 ) |
| Respiratory tract infection | 66 | 2.74 ( 2.15 - 3.49 ) | 2.74 ( 72.59 ) | 2.73 ( 2.23 ) | 1.45 ( 1.1 ) |
| Diverticulitis | 64 | 2.54 ( 1.99 - 3.25 ) | 2.54 ( 59.41 ) | 2.53 ( 2.06 ) | 1.34 ( 0.98 ) |
| Immune system disorder | 60 | 4.84 ( 3.75 - 6.24 ) | 4.83 ( 181.03 ) | 4.8 ( 3.88 ) | 2.26 ( 1.89 ) |
| Kidney infection | 53 | 2.87 ( 2.19 - 3.76 ) | 2.87 ( 64.13 ) | 2.86 ( 2.28 ) | 1.51 ( 1.12 ) |
| Conjunctivitis | 52 | 2.95 ( 2.25 - 3.88 ) | 2.95 ( 66.69 ) | 2.94 ( 2.34 ) | 1.56 ( 1.16 ) |
| Tuberculosis | 52 | 4.54 ( 3.45 - 5.96 ) | 4.54 ( 142.36 ) | 4.51 ( 3.59 ) | 2.17 ( 1.78 ) |
| Gastroenteritis viral | 47 | 2.95 ( 2.22 - 3.93 ) | 2.95 ( 60.38 ) | 2.94 ( 2.31 ) | 1.56 ( 1.14 ) |
| Malignant melanoma | 46 | 3.55 ( 2.66 - 4.74 ) | 3.55 ( 83.71 ) | 3.53 ( 2.77 ) | 1.82 ( 1.4 ) |
| Injection site cellulitis | 44 | 35.4 ( 26.13 - 47.95 ) | 35.37 ( 1394.11 ) | 33.61 ( 26.07 ) | 5.07 ( 4.63 ) |
| Bacterial infection | 43 | 2.72 ( 2.01 - 3.67 ) | 2.72 ( 46.42 ) | 2.71 ( 2.11 ) | 1.44 ( 1 ) |
| Injection site injury | 43 | 8.84 ( 6.54 - 11.95 ) | 8.84 ( 294.82 ) | 8.73 ( 6.79 ) | 3.13 ( 2.69 ) |
| Spinal operation | 43 | 3.22 ( 2.39 - 4.35 ) | 3.22 ( 65.47 ) | 3.21 ( 2.5 ) | 1.68 ( 1.25 ) |
| Eye infection | 42 | 3.92 ( 2.89 - 5.31 ) | 3.91 ( 90.61 ) | 3.9 ( 3.02 ) | 1.96 ( 1.52 ) |
| Mycobacterium tuberculosis complex test positive | 40 | 15.72 ( 11.49 - 21.51 ) | 15.71 ( 538.05 ) | 15.36 ( 11.82 ) | 3.94 ( 3.49 ) |
| Pharyngitis | 35 | 3.16 ( 2.27 - 4.41 ) | 3.16 ( 51.55 ) | 3.15 ( 2.39 ) | 1.66 ( 1.17 ) |
| Pustular psoriasis | 33 | 13.31 ( 9.43 - 18.79 ) | 13.31 ( 368.08 ) | 13.06 ( 9.79 ) | 3.71 ( 3.21 ) |
| Streptococcal infection | 33 | 7.29 ( 5.17 - 10.28 ) | 7.29 ( 177.13 ) | 7.22 ( 5.42 ) | 2.85 ( 2.35 ) |
| Aphthous ulcer | 31 | 3.28 ( 2.3 - 4.67 ) | 3.28 ( 48.83 ) | 3.27 ( 2.43 ) | 1.71 ( 1.2 ) |
| Injection site vesicles | 31 | 6.24 ( 4.38 - 8.88 ) | 6.23 ( 134.95 ) | 6.18 ( 4.6 ) | 2.63 ( 2.12 ) |
| Furuncle | 27 | 3.89 ( 2.66 - 5.68 ) | 3.89 ( 57.57 ) | 3.87 ( 2.82 ) | 1.95 ( 1.41 ) |
| Injection site oedema | 26 | 20 ( 13.54 - 29.55 ) | 19.99 ( 455.13 ) | 19.43 ( 14.01 ) | 4.28 ( 3.72 ) |
| Fear of injection | 25 | 3.48 ( 2.35 - 5.16 ) | 3.48 ( 44 ) | 3.47 ( 2.5 ) | 1.79 ( 1.23 ) |
| Multiple allergies | 25 | 3.4 ( 2.29 - 5.03 ) | 3.4 ( 42.07 ) | 3.38 ( 2.44 ) | 1.76 ( 1.19 ) |
| Tooth abscess | 24 | 2.84 ( 1.9 - 4.24 ) | 2.84 ( 28.46 ) | 2.83 ( 2.02 ) | 1.5 ( 0.92 ) |
| Erysipelas | 24 | 5.23 ( 3.5 - 7.81 ) | 5.23 ( 81.36 ) | 5.19 ( 3.71 ) | 2.38 ( 1.8 ) |
| Tinea infection | 24 | 26.34 ( 17.51 - 39.62 ) | 26.33 ( 562.18 ) | 25.35 ( 18.01 ) | 4.66 ( 4.08 ) |
| Fungal skin infection | 23 | 6.04 ( 4 - 9.1 ) | 6.03 ( 95.73 ) | 5.99 ( 4.25 ) | 2.58 ( 1.99 ) |
| Cardiac operation | 23 | 3.22 ( 2.13 - 4.84 ) | 3.21 ( 34.92 ) | 3.2 ( 2.27 ) | 1.68 ( 1.09 ) |
| Arthropod bite | 22 | 3.44 ( 2.26 - 5.23 ) | 3.44 ( 37.86 ) | 3.43 ( 2.41 ) | 1.78 ( 1.17 ) |
| Injection related reaction | 21 | 11.97 ( 7.77 - 18.43 ) | 11.96 ( 207.17 ) | 11.76 ( 8.2 ) | 3.56 ( 2.94 ) |
| Oral fungal infection | 20 | 9.2 ( 5.92 - 14.31 ) | 9.2 ( 144.16 ) | 9.09 ( 6.28 ) | 3.18 ( 2.55 ) |
| Oesophageal candidiasis | 20 | 6.27 ( 4.03 - 9.73 ) | 6.26 ( 87.65 ) | 6.21 ( 4.3 ) | 2.64 ( 2 ) |
| Antibiotic therapy | 19 | 16.97 ( 10.76 - 26.77 ) | 16.97 ( 278.26 ) | 16.56 ( 11.31 ) | 4.05 ( 3.4 ) |
| Hordeolum | 18 | 4.38 ( 2.76 - 6.97 ) | 4.38 ( 46.69 ) | 4.36 ( 2.96 ) | 2.12 ( 1.46 ) |
| Tonsillitis | 17 | 3.46 ( 2.15 - 5.57 ) | 3.46 ( 29.55 ) | 3.45 ( 2.31 ) | 1.78 ( 1.1 ) |
| Hernia repair | 17 | 8.35 ( 5.18 - 13.48 ) | 8.35 ( 108.6 ) | 8.26 ( 5.53 ) | 3.05 ( 2.36 ) |
| Serum sickness | 16 | 8.58 ( 5.24 - 14.05 ) | 8.58 ( 105.68 ) | 8.48 ( 5.61 ) | 3.08 ( 2.38 ) |
| Injection site scar | 16 | 6.42 ( 3.92 - 10.5 ) | 6.42 ( 72.43 ) | 6.36 ( 4.21 ) | 2.67 ( 1.97 ) |
| Vitiligo | 16 | 7.2 ( 4.4 - 11.79 ) | 7.2 ( 84.54 ) | 7.14 ( 4.72 ) | 2.83 ( 2.13 ) |
| Tinea pedis | 15 | 12.34 ( 7.4 - 20.56 ) | 12.33 ( 153.3 ) | 12.12 ( 7.91 ) | 3.6 ( 2.87 ) |
| Impetigo | 14 | 11.78 ( 6.94 - 19.98 ) | 11.78 ( 135.62 ) | 11.59 ( 7.44 ) | 3.53 ( 2.78 ) |

Abbreviation: ROR, reporting odds ratio; PRR, proportional reporting ratio; EBGM, empirical Bayesian geometric mean; EBGM05, the lower limit of the 95% CI of EBGM; IC, information component; IC025, the lower limit of the 95% CI of the IC; CI, confidence interval; PT, preferred term.

Supplementary Table 6:

Top 35 adverse events with positive signals for IXE at the PT level in males from FAERS data

| PT | Case numbers | ROR(95%CI) | PRR(χ^2^) | EBGM(EBGM05) | IC(IC025) |
| --- | --- | --- | --- | --- | --- |
| Injection site pain | 510 | 7.32 ( 6.64 - 8.08 ) | 7.02 ( 2134.66 ) | 5.84 ( 5.38 ) | 2.55 ( 2.4 ) |
| Injection site erythema | 242 | 15.66 ( 13.38 - 18.32 ) | 15.32 ( 2119.78 ) | 10.35 ( 9.07 ) | 3.37 ( 3.16 ) |
| Injection site swelling | 216 | 16.52 ( 13.97 - 19.54 ) | 16.21 ( 1976.11 ) | 10.73 ( 9.33 ) | 3.42 ( 3.19 ) |
| Injection site reaction | 164 | 18.37 ( 15.1 - 22.36 ) | 18.1 ( 1629.59 ) | 11.5 ( 9.76 ) | 3.52 ( 3.26 ) |
| Injection site mass | 100 | 24.64 ( 18.86 - 32.18 ) | 24.42 ( 1216.39 ) | 13.67 ( 10.94 ) | 3.77 ( 3.43 ) |
| Cellulitis | 86 | 3.08 ( 2.46 - 3.85 ) | 3.06 ( 108.3 ) | 2.86 ( 2.38 ) | 1.52 ( 1.19 ) |
| Injection site pruritus | 79 | 9.31 ( 7.22 - 12.01 ) | 9.25 ( 440.57 ) | 7.25 ( 5.86 ) | 2.86 ( 2.5 ) |
| Injection site rash | 65 | 16.52 ( 12.18 - 22.42 ) | 16.43 ( 600.09 ) | 10.83 ( 8.39 ) | 3.44 ( 3.02 ) |
| Injection site urticaria | 58 | 27.09 ( 18.93 - 38.78 ) | 26.95 ( 749.12 ) | 14.41 ( 10.67 ) | 3.85 ( 3.39 ) |
| Injection site warmth | 50 | 25.39 ( 17.36 - 37.13 ) | 25.27 ( 621.14 ) | 13.93 ( 10.13 ) | 3.8 ( 3.31 ) |
| Ear infection | 50 | 4.35 ( 3.23 - 5.87 ) | 4.34 ( 111.79 ) | 3.9 ( 3.04 ) | 1.96 ( 1.53 ) |
| Fungal infection | 36 | 4.68 ( 3.29 - 6.67 ) | 4.67 ( 89.47 ) | 4.16 ( 3.1 ) | 2.06 ( 1.55 ) |
| Oral candidiasis | 36 | 7.38 ( 5.11 - 10.64 ) | 7.36 ( 157.58 ) | 6.06 ( 4.46 ) | 2.6 ( 2.08 ) |
| Pharyngitis streptococcal | 29 | 5.66 ( 3.8 - 8.43 ) | 5.65 ( 92.74 ) | 4.88 ( 3.5 ) | 2.29 ( 1.72 ) |
| Inflammatory bowel disease | 28 | 12.07 ( 7.76 - 18.77 ) | 12.04 ( 199.97 ) | 8.79 ( 6.07 ) | 3.14 ( 2.52 ) |
| Colitis ulcerative | 23 | 4.17 ( 2.69 - 6.47 ) | 4.17 ( 48.4 ) | 3.77 ( 2.61 ) | 1.91 ( 1.29 ) |
| Candida infection | 22 | 4.57 ( 2.91 - 7.16 ) | 4.56 ( 52.82 ) | 4.07 ( 2.8 ) | 2.03 ( 1.38 ) |
| Colitis | 19 | 3.3 ( 2.05 - 5.31 ) | 3.3 ( 27.31 ) | 3.06 ( 2.06 ) | 1.61 ( 0.93 ) |
| Eye infection | 18 | 6.18 ( 3.71 - 10.29 ) | 6.17 ( 64.29 ) | 5.26 ( 3.43 ) | 2.4 ( 1.67 ) |
| Irritable bowel syndrome | 17 | 4.81 ( 2.88 - 8.04 ) | 4.8 ( 43.88 ) | 4.26 ( 2.77 ) | 2.09 ( 1.36 ) |
| Conjunctivitis | 13 | 5.36 ( 2.96 - 9.68 ) | 5.35 ( 38.79 ) | 4.67 ( 2.84 ) | 2.22 ( 1.39 ) |
| Streptococcal infection | 12 | 6.07 ( 3.26 - 11.32 ) | 6.07 ( 41.94 ) | 5.18 ( 3.08 ) | 2.37 ( 1.5 ) |
| Hernia repair | 10 | 8.74 ( 4.31 - 17.73 ) | 8.73 ( 52.54 ) | 6.93 ( 3.83 ) | 2.79 ( 1.82 ) |
| Injection site cellulitis | 9 | 259.51 ( 32.87 - 2048.63 ) | 259.3 ( 231.58 ) | 26.83 ( 4.76 ) | 4.75 ( 3.48 ) |
| Injection site hypersensitivity | 8 | 32.95 ( 11.95 - 90.88 ) | 32.93 ( 115.58 ) | 15.9 ( 6.8 ) | 3.99 ( 2.79 ) |
| Impetigo | 7 | 8.07 ( 3.49 - 18.67 ) | 8.07 ( 33.86 ) | 6.52 ( 3.23 ) | 2.71 ( 1.57 ) |
| Serum sickness | 7 | 33.63 ( 11.3 - 100.1 ) | 33.61 ( 102.23 ) | 16.05 ( 6.44 ) | 4 ( 2.73 ) |
| Injection related reaction | 7 | 12.61 ( 5.19 - 30.66 ) | 12.6 ( 52.03 ) | 9.07 ( 4.31 ) | 3.18 ( 2 ) |
| Aphthous ulcer | 6 | 5.09 ( 2.14 - 12.12 ) | 5.08 ( 16.73 ) | 4.47 ( 2.16 ) | 2.16 ( 0.98 ) |
| Staphylococcal skin infection | 6 | 6.18 ( 2.56 - 14.92 ) | 6.17 ( 21.43 ) | 5.26 ( 2.52 ) | 2.4 ( 1.2 ) |
| Antibiotic therapy | 6 | 12.35 ( 4.75 - 32.15 ) | 12.35 ( 43.8 ) | 8.94 ( 4.02 ) | 3.16 ( 1.9 ) |
| Pharyngeal swelling | 6 | 5.24 ( 2.2 - 12.51 ) | 5.24 ( 17.41 ) | 4.59 ( 2.21 ) | 2.2 ( 1.01 ) |
| Necrotising fasciitis | 5 | 6 ( 2.29 - 15.74 ) | 6 ( 17.25 ) | 5.14 ( 2.29 ) | 2.36 ( 1.07 ) |
| Miliaria | 5 | 8.01 ( 2.97 - 21.57 ) | 8 ( 23.98 ) | 6.48 ( 2.83 ) | 2.7 ( 1.38 ) |
| Large intestine perforation | 5 | 5.15 ( 1.99 - 13.33 ) | 5.14 ( 14.17 ) | 4.52 ( 2.04 ) | 2.18 ( 0.9 ) |

Abbreviation: ROR, reporting odds ratio; PRR, proportional reporting ratio; EBGM, empirical Bayesian geometric mean; EBGM05, the lower limit of the 95% CI of EBGM; IC, information component; IC025, the lower limit of the 95% CI of the IC; CI, confidence interval; PT,preferred term; AEs, adverse events.

Supplementary Table 7:

Top 35 adverse events with positive signals for IXE at the PT level in females from FAERS data

| PT | Case numbers | ROR(95%CI) | PRR(χ^2^) | EBGM(EBGM05) | IC(IC025) |
| --- | --- | --- | --- | --- | --- |
| Injection site pain | 882 | 6.77 ( 6.28 - 7.29 ) | 6.46 ( 3408.35 ) | 5.53 ( 5.2 ) | 2.47 ( 2.36 ) |
| Injection site erythema | 651 | 13.57 ( 12.37 - 14.89 ) | 13.08 ( 5145.49 ) | 9.52 ( 8.81 ) | 3.25 ( 3.12 ) |
| Injection site swelling | 462 | 16.17 ( 14.44 - 18.1 ) | 15.75 ( 4257.64 ) | 10.82 ( 9.84 ) | 3.44 ( 3.28 ) |
| Injection site reaction | 369 | 15.82 ( 13.95 - 17.94 ) | 15.5 ( 3354.9 ) | 10.7 ( 9.63 ) | 3.42 ( 3.25 ) |
| Injection site pruritus | 254 | 8.56 ( 7.45 - 9.84 ) | 8.45 ( 1316.46 ) | 6.87 ( 6.11 ) | 2.78 ( 2.58 ) |
| Urinary tract infection | 197 | 2.59 ( 2.24 - 3 ) | 2.58 ( 176.34 ) | 2.46 ( 2.17 ) | 1.3 ( 1.08 ) |
| Injection site urticaria | 189 | 17.76 ( 14.85 - 21.24 ) | 17.57 ( 1894.12 ) | 11.62 ( 10 ) | 3.54 ( 3.29 ) |
| Injection site warmth | 164 | 17.58 ( 14.52 - 21.29 ) | 17.42 ( 1632.41 ) | 11.55 ( 9.84 ) | 3.53 ( 3.27 ) |
| Urticaria | 143 | 2.71 ( 2.28 - 3.22 ) | 2.69 ( 140.73 ) | 2.56 ( 2.22 ) | 1.36 ( 1.11 ) |
| Injection site rash | 137 | 13.69 ( 11.19 - 16.74 ) | 13.58 ( 1114.88 ) | 9.78 ( 8.26 ) | 3.29 ( 3.01 ) |
| Illness | 127 | 2.6 ( 2.17 - 3.12 ) | 2.59 ( 114.56 ) | 2.47 ( 2.12 ) | 1.3 ( 1.04 ) |
| Injection site mass | 123 | 15.1 ( 12.17 - 18.73 ) | 14.99 ( 1087.24 ) | 10.46 ( 8.74 ) | 3.39 ( 3.09 ) |
| Ear infection | 105 | 4 ( 3.26 - 4.9 ) | 3.98 ( 208.04 ) | 3.64 ( 3.07 ) | 1.86 ( 1.57 ) |
| Cellulitis | 78 | 2.6 ( 2.06 - 3.28 ) | 2.59 ( 70.54 ) | 2.47 ( 2.03 ) | 1.3 ( 0.97 ) |
| Fungal infection | 75 | 4.47 ( 3.51 - 5.7 ) | 4.46 ( 176.34 ) | 4.03 ( 3.29 ) | 2.01 ( 1.66 ) |
| Injection site induration | 56 | 18.72 ( 13.44 - 26.08 ) | 18.66 ( 586.84 ) | 12.07 ( 9.15 ) | 3.59 ( 3.14 ) |
| Pharyngitis streptococcal | 55 | 4.01 ( 3.03 - 5.31 ) | 4 ( 109.75 ) | 3.66 ( 2.89 ) | 1.87 ( 1.46 ) |
| Tooth infection | 41 | 5.65 ( 4.05 - 7.87 ) | 5.63 ( 132.51 ) | 4.93 ( 3.73 ) | 2.3 ( 1.82 ) |
| Staphylococcal infection | 39 | 2.85 ( 2.05 - 3.95 ) | 2.84 ( 42.71 ) | 2.69 ( 2.04 ) | 1.43 ( 0.95 ) |
| Candida infection | 38 | 4.62 ( 3.29 - 6.5 ) | 4.61 ( 93.82 ) | 4.15 ( 3.12 ) | 2.05 ( 1.56 ) |
| Oral candidiasis | 37 | 4.84 ( 3.42 - 6.84 ) | 4.83 ( 97.41 ) | 4.32 ( 3.23 ) | 2.11 ( 1.61 ) |
| Colitis ulcerative | 32 | 4.11 ( 2.85 - 5.95 ) | 4.11 ( 66.57 ) | 3.75 ( 2.75 ) | 1.91 ( 1.37 ) |
| Overdose | 32 | 6.97 ( 4.75 - 10.23 ) | 6.96 ( 133.72 ) | 5.88 ( 4.26 ) | 2.56 ( 2.01 ) |
| Immune system disorder | 30 | 3.66 ( 2.51 - 5.35 ) | 3.66 ( 51.89 ) | 3.38 ( 2.46 ) | 1.76 ( 1.21 ) |
| Lip swelling | 22 | 4.82 ( 3.08 - 7.56 ) | 4.82 ( 57.74 ) | 4.31 ( 2.96 ) | 2.11 ( 1.47 ) |
| Angioedema | 21 | 5.49 ( 3.45 - 8.73 ) | 5.48 ( 65.52 ) | 4.81 ( 3.27 ) | 2.27 ( 1.61 ) |
| Inflammatory bowel disease | 21 | 10.45 ( 6.38 - 17.14 ) | 10.44 ( 134.51 ) | 8.08 ( 5.35 ) | 3.01 ( 2.32 ) |
| Conjunctivitis | 20 | 3.58 ( 2.26 - 5.69 ) | 3.58 ( 33.39 ) | 3.32 ( 2.25 ) | 1.73 ( 1.07 ) |
| Injection site hypersensitivity | 17 | 15.23 ( 8.53 - 27.19 ) | 15.22 ( 152 ) | 10.57 ( 6.51 ) | 3.4 ( 2.61 ) |
| Injection site cellulitis | 14 | 62.71 ( 25.31 - 155.39 ) | 62.66 ( 283.15 ) | 21.55 ( 10.09 ) | 4.43 ( 3.46 ) |
| Throat tightness | 12 | 3.76 ( 2.07 - 6.85 ) | 3.76 ( 21.71 ) | 3.46 ( 2.1 ) | 1.79 ( 0.95 ) |
| Cardiac operation | 11 | 3.75 ( 2.01 - 7 ) | 3.75 ( 19.78 ) | 3.45 ( 2.05 ) | 1.79 ( 0.91 ) |
| Antibiotic therapy | 11 | 9.32 ( 4.75 - 18.27 ) | 9.31 ( 62.93 ) | 7.41 ( 4.22 ) | 2.89 ( 1.96 ) |
| Injection site injury | 9 | 5.76 ( 2.83 - 11.72 ) | 5.75 ( 29.87 ) | 5.02 ( 2.77 ) | 2.33 ( 1.34 ) |
| Oral fungal infection | 9 | 6.88 ( 3.34 - 14.16 ) | 6.88 ( 37.07 ) | 5.82 ( 3.18 ) | 2.54 ( 1.54 ) |

Abbreviation: ROR, reporting odds ratio; PRR, proportional reporting ratio; EBGM, empirical Bayesian geometric mean; EBGM05, the lower limit of the 95% CI of EBGM; IC, information component; IC025, the lower limit of the 95% CI of the IC; CI, confidence interval; PT,preferred term; AEs, adverse events.

Supplementary Table 8:

Adverse events with positive signals at the PT level for IXE in patients aged under 18 from FAERS data

| PT | Case numbers | ROR(95%CI) | PRR(χ^2^) | EBGM(EBGM05) | IC(IC025) |
| --- | --- | --- | --- | --- | --- |
| Injection site erythema | 12 | 4.8 ( 2.49 - 9.23 ) | 4.66 ( 26.82 ) | 3.82 ( 2.21 ) | 1.93 ( 1.03 ) |
| Injection site swelling | 11 | 7.33 ( 3.56 - 15.08 ) | 7.13 ( 39.97 ) | 5.2 ( 2.84 ) | 2.38 ( 1.41 ) |
| Injection site reaction | 9 | 9.55 ( 4.15 - 21.98 ) | 9.33 ( 42 ) | 6.21 ( 3.09 ) | 2.63 ( 1.54 ) |
| Illness | 8 | 12.71 ( 4.98 - 32.41 ) | 12.44 ( 46.9 ) | 7.35 ( 3.36 ) | 2.88 ( 1.7 ) |
| Injection site rash | 7 | 9.24 ( 3.61 - 23.61 ) | 9.07 ( 31.85 ) | 6.1 ( 2.78 ) | 2.61 ( 1.39 ) |
| Alopecia | 6 | 7.89 ( 2.94 - 21.16 ) | 7.77 ( 23.68 ) | 5.52 ( 2.42 ) | 2.46 ( 1.18 ) |
| Eye infection | 5 | 19.7 ( 5.27 - 73.71 ) | 19.43 ( 38.92 ) | 9.19 ( 3.05 ) | 3.2 ( 1.7 ) |
| Drug hypersensitivity | 4 | 31.45 ( 5.74 - 172.29 ) | 31.1 ( 38.88 ) | 11.03 ( 2.66 ) | 3.46 ( 1.77 ) |
| Vitiligo | 3 | 23.52 ( 3.92 - 141.21 ) | 23.32 ( 25.66 ) | 9.93 ( 2.22 ) | 3.31 ( 1.44 ) |

Abbreviation: ROR, reporting odds ratio; PRR, proportional reporting ratio; EBGM, empirical Bayesian geometric mean; EBGM05, the lower limit of the 95% CI of EBGM; IC, information component; IC025, the lower limit of the 95% CI of the IC; CI, confidence interval; PT, preferred term.

Supplementary Table 9:

Top 35 adverse events with positive signals for IXE at the PT level in patients aged 18 to 64 from FAERS data

| PT | Case numbers | ROR(95%CI) | PRR(χ^2^) | EBGM(EBGM05) | IC(IC025) |
| --- | --- | --- | --- | --- | --- |
| Injection site pain | 496 | 4.18 ( 3.8 - 4.6 ) | 4.07 ( 1015.38 ) | 3.69 ( 3.4 ) | 1.88 ( 1.74 ) |
| Injection site erythema | 381 | 8.85 ( 7.88 - 9.93 ) | 8.63 ( 1982.47 ) | 6.86 ( 6.23 ) | 2.78 ( 2.61 ) |
| Injection site swelling | 299 | 10.5 ( 9.19 - 12 ) | 10.3 ( 1849.92 ) | 7.83 ( 7.01 ) | 2.97 ( 2.78 ) |
| Injection site reaction | 218 | 10.4 ( 8.9 - 12.15 ) | 10.25 ( 1342.34 ) | 7.81 ( 6.86 ) | 2.97 ( 2.74 ) |
| Sinusitis | 191 | 2.98 ( 2.57 - 3.47 ) | 2.96 ( 225.35 ) | 2.77 ( 2.45 ) | 1.47 ( 1.25 ) |
| Injection site pruritus | 167 | 6.84 ( 5.78 - 8.11 ) | 6.77 ( 665.63 ) | 5.67 ( 4.92 ) | 2.5 ( 2.26 ) |
| Illness | 143 | 4.04 ( 3.39 - 4.82 ) | 4.01 ( 284.2 ) | 3.64 ( 3.14 ) | 1.86 ( 1.61 ) |
| Ear infection | 125 | 5.46 ( 4.51 - 6.62 ) | 5.42 ( 379.78 ) | 4.72 ( 4.02 ) | 2.24 ( 1.96 ) |
| Urinary tract infection | 120 | 2.76 ( 2.28 - 3.33 ) | 2.74 ( 121.49 ) | 2.59 ( 2.21 ) | 1.37 ( 1.1 ) |
| Injection site warmth | 119 | 13.81 ( 11.09 - 17.2 ) | 13.7 ( 947.63 ) | 9.58 ( 7.98 ) | 3.26 ( 2.96 ) |
| Injection site mass | 95 | 11.87 ( 9.34 - 15.08 ) | 11.79 ( 664.66 ) | 8.64 ( 7.07 ) | 3.11 ( 2.77 ) |
| Cellulitis | 91 | 3.09 ( 2.49 - 3.84 ) | 3.08 ( 115.54 ) | 2.88 ( 2.4 ) | 1.52 ( 1.21 ) |
| Injection site rash | 87 | 9.87 ( 7.73 - 12.6 ) | 9.82 ( 513.18 ) | 7.56 ( 6.16 ) | 2.92 ( 2.57 ) |
| Fungal infection | 69 | 5.89 ( 4.54 - 7.63 ) | 5.86 ( 231.16 ) | 5.03 ( 4.05 ) | 2.33 ( 1.96 ) |
| Pharyngitis streptococcal | 66 | 5.65 ( 4.33 - 7.36 ) | 5.63 ( 209.9 ) | 4.86 ( 3.9 ) | 2.28 ( 1.9 ) |
| Herpes zoster | 50 | 3.18 ( 2.37 - 4.26 ) | 3.17 ( 67.03 ) | 2.96 ( 2.31 ) | 1.56 ( 1.14 ) |
| Staphylococcal infection | 42 | 2.8 ( 2.04 - 3.85 ) | 2.8 ( 44.16 ) | 2.64 ( 2.02 ) | 1.4 ( 0.94 ) |
| Candida infection | 38 | 5.69 ( 4.02 - 8.07 ) | 5.68 ( 122.31 ) | 4.9 ( 3.66 ) | 2.29 ( 1.79 ) |
| Tooth infection | 38 | 4.94 ( 3.5 - 6.98 ) | 4.93 ( 101.64 ) | 4.35 ( 3.26 ) | 2.12 ( 1.63 ) |
| Respiratory tract infection | 34 | 3.2 ( 2.24 - 4.56 ) | 3.19 ( 46.11 ) | 2.97 ( 2.21 ) | 1.57 ( 1.06 ) |
| Injection site induration | 33 | 11.66 ( 7.77 - 17.48 ) | 11.63 ( 227.94 ) | 8.55 ( 6.1 ) | 3.1 ( 2.53 ) |
| Colitis ulcerative | 30 | 3.47 ( 2.38 - 5.08 ) | 3.47 ( 47.02 ) | 3.2 ( 2.33 ) | 1.68 ( 1.13 ) |
| Oral candidiasis | 27 | 3.71 ( 2.48 - 5.54 ) | 3.71 ( 47.25 ) | 3.4 ( 2.43 ) | 1.76 ( 1.19 ) |
| Colitis | 25 | 3.54 ( 2.33 - 5.36 ) | 3.53 ( 40.44 ) | 3.25 ( 2.3 ) | 1.7 ( 1.1 ) |
| Spinal operation | 25 | 3.65 ( 2.4 - 5.53 ) | 3.64 ( 42.52 ) | 3.34 ( 2.36 ) | 1.74 ( 1.14 ) |
| Immune system disorder | 21 | 3.49 ( 2.22 - 5.49 ) | 3.49 ( 33.2 ) | 3.22 ( 2.2 ) | 1.69 ( 1.04 ) |
| Inflammatory bowel disease | 21 | 9.1 ( 5.57 - 14.87 ) | 9.09 ( 114.65 ) | 7.13 ( 4.73 ) | 2.83 ( 2.15 ) |
| Eye infection | 19 | 4.81 ( 2.96 - 7.82 ) | 4.8 ( 48.96 ) | 4.25 ( 2.83 ) | 2.09 ( 1.4 ) |
| Conjunctivitis | 19 | 3.94 ( 2.44 - 6.36 ) | 3.93 ( 36.52 ) | 3.58 ( 2.39 ) | 1.84 ( 1.15 ) |
| Streptococcal infection | 16 | 3.75 ( 2.23 - 6.31 ) | 3.74 ( 28.47 ) | 3.43 ( 2.21 ) | 1.78 ( 1.03 ) |
| Angioedema | 15 | 3.48 ( 2.04 - 5.96 ) | 3.48 ( 23.66 ) | 3.21 ( 2.05 ) | 1.68 ( 0.92 ) |
| Antibiotic therapy | 15 | 19.49 ( 10.11 - 37.57 ) | 19.47 ( 156.27 ) | 11.98 ( 6.92 ) | 3.58 ( 2.72 ) |
| Cardiac operation | 14 | 4.45 ( 2.53 - 7.81 ) | 4.44 ( 32.32 ) | 3.98 ( 2.48 ) | 1.99 ( 1.2 ) |
| Injection site cellulitis | 13 | 61.92 ( 23.53 - 162.94 ) | 61.87 ( 245.84 ) | 20.22 ( 9 ) | 4.34 ( 3.33 ) |
| Multiple allergies | 11 | 4.99 ( 2.63 - 9.47 ) | 4.99 ( 29.84 ) | 4.39 ( 2.57 ) | 2.14 ( 1.24 ) |

Abbreviation: ROR, reporting odds ratio; PRR, proportional reporting ratio; EBGM, empirical Bayesian geometric mean; EBGM05, the lower limit of the 95% CI of EBGM; IC, information component; IC025, the lower limit of the 95% CI of the IC; CI, confidence interval; PT, preferred term.

Supplementary Table 10:

Adverse events with positive signals at the PT level in patients aged over 64 from FAERS data

| PT | Case numbers | ROR(95%CI) | PRR(χ^2^) | EBGM(EBGM05) | IC(IC025) |
| --- | --- | --- | --- | --- | --- |
| Injection site pain | 91 | 4.98 ( 4 - 6.21 ) | 4.86 ( 252.63 ) | 4.47 ( 3.72 ) | 2.16 ( 1.84 ) |
| Urinary tract infection | 61 | 3.19 ( 2.45 - 4.15 ) | 3.14 ( 83.81 ) | 3 ( 2.41 ) | 1.59 ( 1.2 ) |
| Injection site erythema | 47 | 9.05 ( 6.6 - 12.42 ) | 8.92 ( 275.11 ) | 7.58 ( 5.82 ) | 2.92 ( 2.47 ) |
| Injection site swelling | 39 | 14.25 ( 9.92 - 20.49 ) | 14.07 ( 358.62 ) | 10.89 ( 8.04 ) | 3.44 ( 2.93 ) |
| Sinusitis | 32 | 2.93 ( 2.05 - 4.2 ) | 2.91 ( 37.78 ) | 2.79 ( 2.07 ) | 1.48 ( 0.96 ) |
| Cellulitis | 29 | 3.56 ( 2.44 - 5.21 ) | 3.54 ( 48.96 ) | 3.35 ( 2.43 ) | 1.74 ( 1.19 ) |
| Injection site reaction | 18 | 9.08 ( 5.46 - 15.11 ) | 9.03 ( 106.64 ) | 7.66 ( 5 ) | 2.94 ( 2.22 ) |
| Injection site mass | 16 | 15.61 ( 8.81 - 27.64 ) | 15.52 ( 160.47 ) | 11.71 ( 7.26 ) | 3.55 ( 2.76 ) |
| Injection site pruritus | 16 | 5.57 ( 3.31 - 9.38 ) | 5.54 ( 52.94 ) | 5.03 ( 3.25 ) | 2.33 ( 1.59 ) |
| Injection site warmth | 13 | 19 ( 9.9 - 36.47 ) | 18.92 ( 153.99 ) | 13.5 ( 7.83 ) | 3.76 ( 2.87 ) |
| Ear infection | 13 | 5.64 ( 3.16 - 10.06 ) | 5.62 ( 43.78 ) | 5.09 ( 3.14 ) | 2.35 ( 1.53 ) |
| Fungal infection | 12 | 3.98 ( 2.2 - 7.2 ) | 3.97 ( 24.46 ) | 3.72 ( 2.27 ) | 1.9 ( 1.06 ) |
| Irritable bowel syndrome | 10 | 11.23 ( 5.6 - 22.52 ) | 11.2 ( 73.93 ) | 9.11 ( 5.09 ) | 3.19 ( 2.23 ) |
| Injection site rash | 10 | 8.94 ( 4.52 - 17.66 ) | 8.91 ( 58.35 ) | 7.57 ( 4.28 ) | 2.92 ( 1.97 ) |
| Oral candidiasis | 7 | 5.47 ( 2.49 - 12.01 ) | 5.46 ( 22.67 ) | 4.96 ( 2.57 ) | 2.31 ( 1.23 ) |
| Injection site urticaria | 7 | 13.92 ( 5.94 - 32.62 ) | 13.89 ( 63.55 ) | 10.78 ( 5.29 ) | 3.43 ( 2.28 ) |
| Candida infection | 6 | 5.58 ( 2.39 - 13.07 ) | 5.57 ( 19.98 ) | 5.06 ( 2.48 ) | 2.34 ( 1.18 ) |
| Tooth infection | 6 | 4.69 ( 2.02 - 10.88 ) | 4.68 ( 15.68 ) | 4.32 ( 2.14 ) | 2.11 ( 0.96 ) |
| Skin infection | 6 | 5.25 ( 2.25 - 12.25 ) | 5.24 ( 18.38 ) | 4.79 ( 2.35 ) | 2.26 ( 1.1 ) |
| Injection site induration | 6 | 13.81 ( 5.51 - 34.62 ) | 13.79 ( 54.09 ) | 10.72 ( 4.97 ) | 3.42 ( 2.19 ) |
| Inflammatory bowel disease | 5 | 15.62 ( 5.62 - 43.39 ) | 15.59 ( 50.33 ) | 11.75 ( 5 ) | 3.55 ( 2.21 ) |
| Coronary arterial stent insertion | 5 | 6.07 ( 2.38 - 15.49 ) | 6.06 ( 18.57 ) | 5.45 ( 2.49 ) | 2.45 ( 1.18 ) |
| Bursitis | 5 | 5.33 ( 2.11 - 13.5 ) | 5.32 ( 15.66 ) | 4.85 ( 2.23 ) | 2.28 ( 1.03 ) |
| Device delivery system issue | 5 | 54.67 ( 14.67 - 203.7 ) | 54.58 ( 116.89 ) | 24.81 ( 8.25 ) | 4.63 ( 3.14 ) |
| Epilepsy | 4 | 9.71 ( 3.29 - 28.72 ) | 9.7 ( 25.55 ) | 8.12 ( 3.28 ) | 3.02 ( 1.6 ) |
| Colitis microscopic | 3 | 14.57 ( 3.94 - 53.84 ) | 14.55 ( 28.4 ) | 11.17 ( 3.74 ) | 3.48 ( 1.83 ) |
| Injection related reaction | 3 | 32.78 ( 7.33 - 146.53 ) | 32.75 ( 52.76 ) | 19.14 ( 5.47 ) | 4.26 ( 2.49 ) |
| Guillain-barre syndrome | 3 | 8.74 ( 2.53 - 30.21 ) | 8.73 ( 17.12 ) | 7.44 ( 2.64 ) | 3.26 ( 1.63 ) |

Abbreviation: ROR, reporting odds ratio; PRR, proportional reporting ratio; EBGM, empirical Bayesian geometric mean; EBGM05, the lower limit of the 95% CI of EBGM; IC, information component; IC025, the lower limit of the 95% CI of the IC; CI, confidence interval; PT, preferred term.

Supplementary Table 11:

Top 45 adverse events with positive signals for IXE excluding common medication co-usage at the PT level from FAERS data

| PT | Case numbers | ROR(95%CI) | PRR(χ^2^) | EBGM(EBGM05) | IC(IC025) |
| --- | --- | --- | --- | --- | --- |
| Injection site pain | 3,567 | 16.23 ( 15.69 - 16.8 ) | 15.22 ( 46542.79 ) | 14.9 ( 14.48 ) | 3.9 ( 3.85 ) |
| Injection site erythema | 2,003 | 26.43 ( 25.26 - 27.66 ) | 25.49 ( 45444.99 ) | 24.58 ( 23.66 ) | 4.62 ( 4.55 ) |
| Injection site swelling | 1,510 | 28.54 ( 27.09 - 30.07 ) | 27.76 ( 37429.27 ) | 26.69 ( 25.55 ) | 4.74 ( 4.66 ) |
| Injection site reaction | 1,449 | 30.63 ( 29.04 - 32.31 ) | 29.83 ( 38676.84 ) | 28.59 ( 27.34 ) | 4.84 ( 4.76 ) |
| Injection site pruritus | 689 | 14.58 ( 13.51 - 15.73 ) | 14.4 ( 8417.7 ) | 14.12 ( 13.25 ) | 3.82 ( 3.71 ) |
| Injection site urticaria | 604 | 32.86 ( 30.27 - 35.67 ) | 32.5 ( 17583.25 ) | 31.02 ( 28.96 ) | 4.96 ( 4.83 ) |
| Injection site mass | 547 | 15.34 ( 14.09 - 16.71 ) | 15.2 ( 7096.87 ) | 14.88 ( 13.85 ) | 3.9 ( 3.77 ) |
| Injection site haemorrhage | 511 | 7.88 ( 7.22 - 8.61 ) | 7.82 ( 3007.35 ) | 7.74 ( 7.19 ) | 2.95 ( 2.82 ) |
| Nasopharyngitis | 474 | 2.84 ( 2.6 - 3.11 ) | 2.83 ( 558.33 ) | 2.82 ( 2.61 ) | 1.49 ( 1.36 ) |
| Injection site rash | 464 | 20.55 ( 18.73 - 22.55 ) | 20.38 ( 8300.39 ) | 19.8 ( 18.32 ) | 4.31 ( 4.17 ) |
| Injection site warmth | 445 | 38.51 ( 34.99 - 42.39 ) | 38.2 ( 15246.37 ) | 36.17 ( 33.38 ) | 5.18 ( 5.04 ) |
| Urticaria | 427 | 3.1 ( 2.81 - 3.41 ) | 3.08 ( 598.65 ) | 3.07 ( 2.83 ) | 1.62 ( 1.48 ) |
| Injection site bruising | 414 | 6.74 ( 6.12 - 7.43 ) | 6.7 ( 1989.68 ) | 6.64 ( 6.12 ) | 2.73 ( 2.59 ) |
| Sinusitis | 396 | 4.39 ( 3.98 - 4.85 ) | 4.36 ( 1022.11 ) | 4.34 ( 4 ) | 2.12 ( 1.97 ) |
| Illness | 311 | 2.95 ( 2.64 - 3.3 ) | 2.94 ( 396.92 ) | 2.93 ( 2.67 ) | 1.55 ( 1.39 ) |
| Influenza | 290 | 2.83 ( 2.52 - 3.17 ) | 2.82 ( 339.08 ) | 2.81 ( 2.55 ) | 1.49 ( 1.32 ) |
| Cellulitis | 251 | 5.93 ( 5.24 - 6.72 ) | 5.91 ( 1015.61 ) | 5.87 ( 5.29 ) | 2.55 ( 2.37 ) |
| Ear infection | 211 | 8.7 ( 7.59 - 9.97 ) | 8.67 ( 1413.64 ) | 8.57 ( 7.65 ) | 3.1 ( 2.9 ) |
| Upper respiratory tract infection | 183 | 4.53 ( 3.91 - 5.24 ) | 4.51 ( 497.57 ) | 4.49 ( 3.97 ) | 2.17 ( 1.95 ) |
| Bronchitis | 174 | 2.79 ( 2.4 - 3.24 ) | 2.78 ( 198.19 ) | 2.78 ( 2.45 ) | 1.47 ( 1.25 ) |
| Fungal infection | 172 | 5.92 ( 5.1 - 6.88 ) | 5.91 ( 695.19 ) | 5.86 ( 5.17 ) | 2.55 ( 2.33 ) |
| Injection site induration | 156 | 17.55 ( 14.96 - 20.57 ) | 17.5 ( 2364.5 ) | 17.07 ( 14.94 ) | 4.09 ( 3.86 ) |
| Herpes zoster | 133 | 2.53 ( 2.14 - 3 ) | 2.53 ( 122.71 ) | 2.52 ( 2.19 ) | 1.34 ( 1.09 ) |
| Oral candidiasis | 133 | 13.03 ( 10.97 - 15.47 ) | 13 ( 1444.7 ) | 12.77 ( 11.06 ) | 3.67 ( 3.42 ) |
| Candida infection | 125 | 7.34 ( 6.16 - 8.76 ) | 7.33 ( 675.95 ) | 7.26 ( 6.26 ) | 2.86 ( 2.6 ) |
| Pharyngitis streptococcal | 111 | 11.62 ( 9.63 - 14.03 ) | 11.6 ( 1057.02 ) | 11.42 ( 9.76 ) | 3.51 ( 3.24 ) |
| Inflammatory bowel disease | 96 | 20.28 ( 16.55 - 24.85 ) | 20.24 ( 1704.16 ) | 19.67 ( 16.6 ) | 4.3 ( 4 ) |
| Colitis | 94 | 2.76 ( 2.25 - 3.38 ) | 2.76 ( 104.86 ) | 2.75 ( 2.32 ) | 1.46 ( 1.16 ) |
| Staphylococcal infection | 91 | 3.8 ( 3.09 - 4.67 ) | 3.79 ( 186.24 ) | 3.78 ( 3.18 ) | 1.92 ( 1.62 ) |
| Irritable bowel syndrome | 81 | 4.6 ( 3.7 - 5.73 ) | 4.6 ( 226.37 ) | 4.57 ( 3.81 ) | 2.19 ( 1.87 ) |
| Tooth infection | 79 | 6.71 ( 5.38 - 8.38 ) | 6.71 ( 379.77 ) | 6.65 ( 5.52 ) | 2.73 ( 2.41 ) |
| Viral infection | 70 | 2.52 ( 1.99 - 3.19 ) | 2.52 ( 64.02 ) | 2.52 ( 2.07 ) | 1.33 ( 0.99 ) |
| Respiratory tract infection | 66 | 2.78 ( 2.18 - 3.54 ) | 2.78 ( 74.88 ) | 2.77 ( 2.26 ) | 1.47 ( 1.12 ) |
| Diverticulitis | 62 | 2.5 ( 1.94 - 3.2 ) | 2.49 ( 55.29 ) | 2.49 ( 2.02 ) | 1.32 ( 0.95 ) |
| Injection site hypersensitivity | 59 | 22.76 ( 17.55 - 29.5 ) | 22.73 ( 1185.21 ) | 22.01 ( 17.71 ) | 4.46 ( 4.08 ) |
| Immune system disorder | 58 | 4.74 ( 3.66 - 6.14 ) | 4.74 ( 169.87 ) | 4.71 ( 3.8 ) | 2.24 ( 1.86 ) |
| Kidney infection | 53 | 2.91 ( 2.22 - 3.81 ) | 2.91 ( 66.08 ) | 2.9 ( 2.31 ) | 1.54 ( 1.14 ) |
| Conjunctivitis | 52 | 2.99 ( 2.28 - 3.93 ) | 2.99 ( 68.68 ) | 2.98 ( 2.37 ) | 1.58 ( 1.18 ) |
| Tuberculosis | 51 | 4.52 ( 3.43 - 5.95 ) | 4.51 ( 138.54 ) | 4.49 ( 3.56 ) | 2.17 ( 1.76 ) |
| Gastroenteritis viral | 47 | 3 ( 2.25 - 3.99 ) | 2.99 ( 62.17 ) | 2.99 ( 2.35 ) | 1.58 ( 1.16 ) |
| Ankylosing spondylitis | 47 | 3.54 ( 2.65 - 4.71 ) | 3.53 ( 84.98 ) | 3.52 ( 2.77 ) | 1.82 ( 1.4 ) |
| Malignant melanoma | 45 | 3.52 ( 2.63 - 4.72 ) | 3.52 ( 80.8 ) | 3.51 ( 2.74 ) | 1.81 ( 1.38 ) |
| Injection site cellulitis | 43 | 35.06 ( 25.8 - 47.65 ) | 35.03 ( 1350.19 ) | 33.32 ( 25.78 ) | 5.06 ( 4.61 ) |
| Bacterial infection | 43 | 2.76 ( 2.04 - 3.72 ) | 2.76 ( 47.89 ) | 2.75 ( 2.14 ) | 1.46 ( 1.02 ) |
| Injection site injury | 43 | 8.97 ( 6.64 - 12.12 ) | 8.96 ( 300.24 ) | 8.86 ( 6.89 ) | 3.15 ( 2.71 ) |

Abbreviation: ROR, reporting odds ratio; PRR, proportional reporting ratio; EBGM, empirical Bayesian geometric mean; EBGM05, the lower limit of the 95% CI of EBGM; IC, information component; IC025, the lower limit of the 95% CI of the IC; CI, confidence interval; PT, preferred term.
